# Soil phosphorus status and P nutrition strategies of European beech forests on carbonate compared to silicate parent material

**DOI:** 10.1007/s10533-021-00884-7

**Published:** 2022-02-02

**Authors:** Jörg Prietzel, Jaane Krüger, Klaus Kaiser, Wulf Amelung, Sara L. Bauke, Michaela A. Dippold, Ellen Kandeler, Wantana Klysubun, Hans Lewandowski, Sebastian Löppmann, Jörg Luster, Sven Marhan, Heike Puhlmann, Marius Schmitt, Maja B. Siegenthaler, Jan Siemens, Sandra Spielvogel, Sabine Willbold, Jan Wolff, Friederike Lang

**Affiliations:** 1 Chair of Soil Science, School of Life Sciences Weihenstephan, Technical University Munich, Emil-Ramann-Str. 2, 85354 Freising, Germany; 2 Professur für Bodenökologie, Albert-Ludwigs-Universität Freiburg, Bertoldstr. 17, 79085 Freiburg, Germany; 3 Soil Sciences, Martin Luther University Halle Wittenberg, Von-Seckendorff-Platz 3, 06120 Halle (Saale), Germany; 4 Institute für Nutzpflanzenwissenschaften und Ressourcenschutz (INRES), Allgemeine Bodenkunde und Bodenökologie, Universität Bonn, Nussallee 13, 53115 Bonn, Germany; 5 Biogeochemie der Agrarökosysteme, Georg-August-Universität Göttingen, Büsgenweg 2, 37077 Göttingen, Germany; 6 Institut für Bodenkunde und Standortslehre, Fachgebiet Bodenbiologie, Universität Hohenheim, Emil-Wolff-Str. 27, 70593 Stuttgart, Germany; 7 Synchrotron Light Research Institute, 111 Moo 6 University Avenue, Muang District, Nakhon Ratchasima, 30000 Thailand; 8 Institut für Bio- und Geowissenschaften – IBG-3: Agrosphäre, Forschungszentrum Jülich GmbH, Wilhelm-Johnen-Straße, 52428 Jülich, Germany; 9 Institut für Pflanzenernährung und Bodenkunde, Christian-Albrechts-Universität zu Kiel, Abteilung Bodenkunde, Hermann-Rodewaldstr. 2, 24118 Kiel, Germany; 10 Forest Soils and Biogeochemistry, Swiss Federal Research Institute WSL, 8903 Birmensdorf, Switzerland; 11 Forstliche Versuchs- und Forschungsanstalt Baden-Württemberg, Wonnhaldestr. 4, 79100 Freiburg, Germany; 12 Institute of Agricultural Sciences, ETH Zurich, Eschikon 33, 8315 Lindau, Switzerland; 13 Professur für Bodenressourcen und Bodenschutz, Institut für Bodenkunde und Bodenerhaltung, Interdisziplinäres Forschungszentrum (iFZ), Justus-Liebig-Universität Giessen, Heinrich-Buff-Ring 26-32, 35392 Gießen, Germany

**Keywords:** Calcareous soils, Ecosystem nutrition, Soil P forms, Pedogenesis, Bedrock impurity, P acquiring, P recycling

## Abstract

Sustainable forest management requires understanding of ecosystem phosphorus (P) cycling. Lang et al. (2017) [*Biogeochemistry,* https://doi.org/10.1007/s10533-017-0375-0] introduced the concept of P-acquiring *vs.* P-recycling nutrition strategies for European beech (*Fagus sylvatica L.*) forests on silicate parent material, and demonstrated a change from P-acquiring to P-recycling nutrition from P-rich to P-poor sites. The present study extends this silicate rock-based assessment to forest sites with soils formed from carbonate bedrock. For all sites, it presents a large set of general soil and bedrock chemistry data. It thoroughly describes the soil P status and generates a comprehensive concept on forest ecosystem P nutrition covering the majority of Central European forest soils. For this purpose, an Ecosystem P Nutrition Index (*ENI*_*P*_) was developed, which enabled the comparison of forest P nutrition strategies at the carbonate sites in our study among each other and also with those of the silicate sites investigated by Lang et al. (2017). The P status of forest soils on carbonate substrates was characterized by low soil P stocks and a large fraction of organic Ca-bound P (probably largely Ca phytate) during early stages of pedogenesis. Soil P stocks, particularly those in the mineral soil and of inorganic P forms, including Al- and Fe-bound P, became more abundant with progressing pedogenesis and accumulation of carbonate rock dissolution residue. Phosphorus-rich impure, silicate-enriched carbonate bedrock promoted the accumulation of dissolution residue and supported larger soil P stocks, mainly bound to Fe and Al minerals. In carbonate-derived soils, only low P amounts were bioavailable during early stages of pedogenesis, and, similar to P-poor silicate sites, P nutrition of beech forests depended on tight (re)cycling of P bound in forest floor soil organic matter (SOM). In contrast to P-poor silicate sites, where the ecosystem P nutrition strategy is direct biotic recycling of SOM-bound organic P, recycling during early stages of pedogenesis on carbonate substrates also involves the dissolution of stable Ca-P_org_ precipitates formed from phosphate released during SOM decomposition. In contrast to silicate sites, progressing pedogenesis and accumulation of P-enriched carbonate bedrock dissolution residue at the carbonate sites promote again P-acquiring mechanisms for ecosystem P nutrition.

**Supplementary Information:**

The online version contains supplementary material available at 10.1007/s10533-021-00884-7.

## Introduction

Several recent studies (*e.g.* Prietzel and Stetter 2010; Talkner et al. 2015; Jonard et al. 2015; Prietzel et al. 2020) reported increasing phosphorus (P) limitation of European forests. These trends highlight the need for improved understanding of ecosystem P nutrition strategies to support the development of P-sustainable forest management. For forests on siliceous substrates, Lang et al. (2016) proposed the concept of ecosystems acquiring P on sites with sufficient lithogenic P sources (apatite) *vs.* P-recycling ecosystems characterized by P bound to soil organic matter (SOM) at sites with little lithogenic P in the rooting zone. The usefulness of the approach was highlighted by Lang et al. (2017), who identified parameters allowing for rating the relative contribution of the two strategies for P nutrition in temperate forest ecosystems on silicate parent material. Phosphorus-acquiring ecosystems had larger soil P stocks and accumulated moderately labile P in topsoil horizons. With decreasing soil P stocks and increasing relevance of ecosystem P recycling, forest floor turnover rates decreased, while C/P ratios in the Oa and A horizons increased. Moreover, P in fine-root biomass increased relative to microbial-bound P. High proportions of fine-root biomass in forest floors seemed to favor tight P recycling. Intense P recycling improved the P use efficiency of beech forests on silicate parent material.

Forests on soils formed from carbonate bedrock often feature pronounced P limitation. This is particularly true where the parent material is low in P (Porder and Ramachandran 2013), and where soils are at an early stage of pedogenesis (*e.g.* Rendzic Leptosols) with pH values > 6.5 and carbonate in the entire profile (Baier et al. 2006; Prietzel and Ammer 2008; Prietzel et al. 2015). Current assumptions on reasons for the poor P nutrition of forests on carbonate sites follow several lines of argument. Soil P contents are generally smaller in shallow, stony Rendzic Leptosols than in most soils developed from silicate parent material (Schubert 2002; Prietzel et al. 2015). Furthermore, orthophosphate (oPO_4_) released by chemical weathering of lithogenic carbonate-entrapped apatite [Ca_5_(OH)(PO_4_)_3_] immediately re-precipitates most often as sparsely soluble secondary Ca-PO_4_ minerals (Hinsinger 2001) and/or is strongly adsorbed to carbonate mineral surfaces (Wan et al. 2016). In principle, when access to inorganic P is limited, enzymatic cleavage of SOM-bound organic P (P_org_) is an important process of plant P acquisition (Hinsinger 2001), also known for N (*e.g.* Turner et al. 2014). However, even though P mineralization rates are potentially high at elevated pH values, there is increasing evidence (McKercher and Anderson 1989; Celi et al. 2000; Crea et al. 2006; Celi and Barberis 2007; Wan et al. 2016; Prietzel et al. 2016a) that not only oPO_4_, but also several dissolved organic P (DOP) forms like inositol P strongly bind to the abundant Ca^2+^ ions in the soil solution, soil matrix, and bedrock. Overall, sparsely soluble und thus poorly bioavailable Ca-bound organic P (*e.g*. Ca inositol phosphates [“Ca phytate”] and inositol phosphates adsorbed to carbonate rock surfaces, Celi et al. 2000) are major P forms in temperate carbonate forest soils (Prietzel et al. 2016b).

Walker and Syers (1976) developed a fundamental concept how stocks of total P and different P forms in soils systematically change with pedogenesis, affecting ecosystem P supply. According to this concept, P from bedrock-bound primary minerals (mostly apatite) is transformed into P_org_ and P bound to secondary minerals at initial stages of pedogenesis. This transformation is associated with continuous soil and ecosystem P losses. With progressing pedogenesis and ecosystem maturation, P mobilization from primary minerals in the rooted zone of soils is becoming increasingly irrelevant and replaced by transformation of the remaining soil P into hardly-bioavailable organic and/or secondary mineral-bound soil P forms. During this process, the native ecosystem slowly changes from N to P limitation. The concept originally had been developed for temperate-humid soils on silicate substrate in New Zealand. Later, it has been proven true also for silicate soils under cool-humid (*e.g.* Giguet-Covex et al. 2013; Prietzel et al. 2013), semiarid (Selmants and Hart 2010), and subtropical climate (Chen et al. 2015). Furthermore, it has been modified for soils subject to high aeolic P input (Heindel et al. 2017; Gu et al. 2019), and for soils under cold polar climate (Prietzel et al. 2019). According to these studies, depending on site conditions, the time of change in soil P forms as predicted by the Walker and Syers (1976) model may vary between only decades for forests under temperate moist climate (Prietzel et al. 2013; Turner et al. 2013) up to several million years for grasslands under semi-arid climate (Selmants and Hart 2010). However, so far, no study on pedogenesis effects on the P status of soils derived from carbonate parent material has been published, and it remains unclear whether the concept of Walker and Syers (1976) is also valid for carbonate soils. In this study, we investigated forest soils developed from carbonate parent material using the same set of variables as in the study on silicate soils by Lang et al. (2017). We assume that (1) the poor P status of forests on carbonate parent material is at least partly caused by the small P stocks and/or peculiar P speciation of their soils (Prietzel et al. 2015; 2016b), and that (2) in contrast to initial silicate soils (Crews et al. 1995; Wardle et al. 2004), initial carbonate soils often show particularly poor P supply (Ewald, 2000; Prietzel and Ammer, 2008; Prietzel et al. 2015). Based on the overall assumption that the P nutrition strategy of beech forest ecosystems is controled by the P supply of soils, we further assume that (3) the concept for P-acquiring and recycling and the indicators can also be applied to soils developing from carbonate parent material. We thus addressed the following hypotheses:

- The concept of Walker and Syers (1976) describing changes of soil P stocks and P forms with progressing pedogenesis is also valid for soils formed on carbonate parent material.
- Temperate forest soils formed from carbonate substrate differ from those formed from silicate parent material in terms of stocks and P speciation: The soils from carbonate bedrock are characterized by generally smaller total P stocks, and predominance of sparsely soluble Ca-bound organic P, resulting in poor ecosystem P availability and high relevance of soil P_org_ turnover for ecosystem P nutrition.
- The concept of P-acquiring *vs.* P-recycling ecosystems developed for temperate forests at silicate sites by Lang et al. (2016; 2017) is also applicable for carbonate sites.

## Materials and methods

### Study sites

The study was conducted at four sites with European beech (*Fagus sylvatica L.*)-dominated forests on soils developed from different carbonate parent materials (dolostone, limestone) and different stages of pedogenesis. Site *Mangfallgebirge* (*MAN*; 47°36’N, 11°49’E) is located in the German Limestone Alps. It consists of two beech-dominated mixed mountain forest (*F. sylvatica, Picea abies, Abies alba, Acer pseudoplatanus*) stands, one covering the N-exposed and one the opposing S-exposed slope of the Lange Au valley. The parent material of soil formation is dolostone. Site *Tuttlingen* (*TUT*; 47°59’N, 8°45’E) is located in the Swabian Alb (SW Germany). It consists of two mature beech forest stands, one covering the NE-exposed and one the opposing SW-exposed slope of the Krähenbach valley. The parent material of soil formation is limestone. At *MAN* (Biermayer and Rehfuess 1985) and also at *TUT*, pedogenesis started after the end of the last glaciation, *i.e.* about 12,000 years ago; pre-Pleistocene soils had been removed by periglacial solifluction. The third site *Bärenthal* (*BAE*; 48°4’N, 8°55'E) is located on a plateau in the Swabian Alb at 16 km distance to *TUT*. It consists of a mature beech forest with admixed *A. pseudoplatanus, A. alba,* and *P. abies*. The parent material is also limestone. The flat topography has resulted in conservation of soil material formed by intensive chemical weathering during the Neogene (Stahr and Böcker 2014). The soils at *TUT SW* (shallow Rendzic Leptosol), *TUT NE* (Rendzic Leptosol with more advanced pedogenesis and a BA horizon), and *BAE* (Cambisol with thick B horizon) are located within 16 km distance from each other. They have similar parent material, climate, and forest vegetation (Table 1), but represent a series of progressing pedogenesis. The fourth site is *Schänis* (*SCH*; 47°09’N; 9°02’E; start of pedogenesis also about 12,000 years ago), located in the Swiss Alps in Canton St. Gallen at a W-exposed slope. It is covered by mature mountain forest dominated by *F. sylvatica.* The climatic conditions are more humid and cool than at *TUT,* but drier and warmer than at *MAN*. The bedrock at *SCH* are Neogene sediments and carbonate conglomerate. Detailed information on all study sites, their soils, and bedrock chemistry is presented in Tables 1, 2 and Table S1 in the Supplementary Information. Note that despite high carbonate contents in bedrock (Table S1), in the weathered soil A horizons the contents of Al and Fe exceeded those of Mg and Ca, pointing to clays and oxyhydroxides as bonding sites for P apart from cation bridges. Briefly, all sites had carbonate parent material, but differed in (i) carbonate type and purity (dolostone at *MAN*, limestone at *TUT* and *BAE*, mixture of limestone and silicate parent material at *SCH*), (ii) time of pedogenesis (limestone site *TUT* << *BAE*), microclimate (N-exposed sites at *MAN* and *TUT* were cooler and moister than their S-exposed counterparts), and bedrock P content (*TUT SW* < *TUT NE*).

**Table 1:** Important site and stand properties of the study sites *Mangfallgebirge*, *Tuttlingen*, *Bärenthal,* and *Schänis*

	Mangfallgebirge N		Mangfallgebirge S		Tuttlingen NE		Tuttlingen SW		Bärenthal		Schänis
Location	Bavarian Limestone Alps				––––––––––––- Swabian Alb ––––––––––––––-						Swiss Alps
Elevation [m a.s.l]	––––––––– 1000 ––––––––-				––––––––- 790 –––––––––			750			730
Aspect	North		South		North-east		South-west	None (plateau)			West
Slope inclination [°]	––––––––- 35 –––––––-				–––––––– 25 ––––––––			2			30
Bedrock	Triassic dolostone (Norian “Hauptdolomit”)				Jurassic limestone (Oxfordian)			Jurassic limestone (Kimmeridgian)			Tertiary sediments Carbonate conglomerate
Bedrock P content [mg g^−1^]	0.14		0.15		0.65		0.14	0.15			0.27
Bedrock carbonate content [mg g^−1^]	996		950		960		985	949			518
Dominating soil types (WRB, 2015)	–––––––– Rendzic Leptosol (Endoleptic Cambisol) ––––––––––––-							Endoleptic Cambisol			Eutric Cambisol
MAT [°C]											
Long-term average*	–––––––––- 6.5 –––––––––-			–––– 6.6 (2002: 14.3)* –––––-				6.6*			7.0
Site	2017/18: 6.8		2017/18: 7.3	––––––- 2002: 13.9 –––––––				ND			7.0
Topsoil Temp. (5 cm)	2017/18: 6.8		2017/18: 7.6	2002: 12.2			2002: 13.2	ND			ND
MAP [mm]											
Long-term average*	–––––––––- 1995 –––––––––			––––– 856 (2002: 551)* –––––				856*			1965
Site	–––––- 2018/19: 2150** ––––––			2002: 602			2002: 784	ND			1965
Stand age [yr]	––––––––- 200–300 ––––––––			––––––––- 85–95 ––––––––				Mature			150
Tree species composition	*Fagus sylvatica, Picea abies,* *Abies alba, Acer pseudoplatanus*			*Fagus sylvatica (*> *90% basal area)* *(Picea abies, Acer pseudoplatanus)*				*Fagus sylvatica (Fraxinus excelsior, Acer pseudoplatanus, Abies alba, Picea abies)*			
Basal area [m^2^ ha^−1^]	35	45		27		20		ND			49
Foliar P (beech) [mg g^−1^]	1.28	1.43		1.18		1.06		ND			1.13
Litterfall [kg ha^−1^ yr^−1^]	2946**	4745**		2019: 6266***		ND		ND		4347	
Litterfall P [kg ha^−1^ yr^−1^]	1.73**	1.39**		2019: 2.73***		ND		ND		3.69	
Data source	**Kohlpaintner and Göttlein (2020), otherwise own data			Nahm et al. (2006) ***calculated from data received by H. Puhlmann (pers. Comm)							

^*^Nearby station *Tuttlingen* of German Meteorological Service

**Table 2:** Major chemical properties of the soils at the study sites (ND = not determined)

Site	Hori-zon	Depth (cm)	pH KCl	ECEC µmol_c_ g^−1^	BS %	Total C (mg g^−1^)	Inorganic C (mg g^−1^)	Total N (mg g^−1^)	Total P (mg g^−1^)	Total Ca (mg g^−1^)	Total Mg (mg g^−1^)	Total K (mg g^−1^)	Total Na (mg g^−1^)	Total Fe (mg g^−1^)	Total Al (mg g^−1^)	Fe dith (mg g^−1^)	Fe oxal (mg g^−1^)	Al oxal (mg g^−1^)
*Mangfall-*	Oi	14–12	5.6	ND	ND	508.9	0	14.5	0.44	19.0	3.8	1.5	0.1	1.2	2.0	ND	ND	ND
*gebirge S1*	Oe	12–4	5.7	901	100	496.0	0	19.3	0.79	22.5	6.5	4.1	0.2	4.9	8.9	ND	ND	ND
	Oa	4–0	6.2	1065	99	411.4	0	18.5	0.86	26.6	11.6	8.7	0.6	12.4	22.9	ND	ND	ND
	Ah1	0–4	6.5	1074	99	246.4	0	13.8	0.82	36.3	21.6	16.3	1.0	23.9	49.6	16.0	6.7	5.9
	Ah2	4–9	6.6	1011	97	206.3	0	12.2	0.74	31.7	20.3	20.3	1.5	27.5	56.6	18.2	8.2	6.7
	Ah3	9–15	6.8	955	99	167.0	0	10.5	0.67	30.9	22.0	20.4	1.2	32.3	68.5	20.4	9.1	7.1
	Ah4	15–22	6.8	922	100	136.1	0	8.6	0.61	26.7	21.8	22.7	1.4	37.2	76.4	23.0	9.9	7.5
	Bk	22–37	6.6	668	100	66.3	2.0	4.5	0.40	15.6	19.6	26.4	1.8	50.9	91.7	28.4	10.9	7.5
*Mangfall-*	Oi	27–23	5.1	ND	ND	508.0	0	16.2	0.43	18.6	2.2	1.3	0.0	0.8	1.1	ND	ND	ND
*gebirge S2*	Oe	23–17	5.2	762	99	489.0	0	22.4	0.89	21.2	3.9	2.8	0.1	3.2	5.8	ND	ND	ND
	Oa	17–0	5.3	894	99	349.0	0	18.4	0.91	25.0	10.1	9.1	1.0	15.5	31.3	ND	ND	ND
	Ah	0–13	6.7	974	98	175.5	10.3	10.9	0.86	37.8	22.8	15.3	2.2	26.4	60.5	16.2	11.1	12.3
	CA	13–30	7.1	706	99	114.8	26.1	7.7	0.84	80.5	43.5	14.5	1.9	24.4	47.4	14.5	9.7	10.5
*Mangfall-*	Oi	15–13	5.1	ND	ND	526.8	0	16.1	0.56	18.7	2.2	1.1	0.1	0.3	0.3	ND	ND	ND
*gebirge N1*	Oe	13–10	5.9	1019	99	526.6	0	23.0	0.78	16.9	3.0	1.3	0.1	1.1	1.6	ND	ND	ND
	Oa	10–0	6.1	1239	97	422.2	0	23.1	1.16	47.9	21.4	3.1	0.4	5.2	9.2	ND	ND	ND
	Ah	0–9	6.8	897	98	243.7	0.0	14.8	1.32	132	76.3	3.9	0.7	6.8	13.9	4.5	3.7	6.0
	CA	9–20	7.4	317	96	95.1	59.6	5.7	0.89	186	115	2.8	0.6	4.7	9.7	3.7	2.5	4.2
*Mangfall-*	Oi	11–10	4.7	ND	ND	516.9	0	13.6	0.46	16.9	1.8	1.3	0.0	0.6	0.3	ND	ND	ND
*gebirge N2*	Oe	10–7	5.1	603	97	512.7	0	20.9	0.79	18.0	4.0	1.5	0.1	1.8	2.2	ND	ND	ND
	Oa	7–0	5.9	1091	100	407.5	0	22.9	1.37	46.1	18.9	4.2	0.4	7.2	13.6	ND	ND	ND
	Ah	0–21	6.8	917	96	287.9	0	17.4	1.58	97.1	45.8	7.5	1.0	10.0	19.6	4.3	4.3	8.0
	CA	21–43	7.4	425	98	124.9	40.5	7.8	1.21	169	103	4.6	0.9	8.3	16.6	3.8	3.7	6.9

Site	Horizon	Depth Cm	pH KCl	ECEC mmol_c_ g^−1^	BS %	Total C (mg g^−1^)	Inorganic C (mg g^−1^)	Total N (mg g^−1^)	Total P (mg g^−1^)	Total Ca (mg g^−1^)	Total Mg (mg g^−1^)	Total K (mg g^−1^ )	Total Na (mg g^−1^)	Total Fe (mg g^−1^)	Total Al (mg g^−1^)	Fe dith (mg g^−1^)	Fe oxal (mg g^−1^)	Al oxal (mg g^−1^)
*Tuttlingen*	Oi	8–4	5.6	ND	ND	483.4	0	13.4	0.70	23.2	1.4	1.8	0.4	0.8	1.1	ND	ND	ND
*NE*	Oe	4–0	6.1	560	100	350.4	0	17.9	1.02	21.8	3.2	6.7	2.4	12.1	20.1	ND	ND	ND
	Ah1	0–8	6.1	560	98	101.4	0.0	7.1	1.00	11.8	5.9	14.2	5.7	32.0	64.4	24.0	2.9	4.9
	Ah2	8–14	6.4	494	99	76.7	0.0	5.8	1.03	11.6	6.1	14.3	6.0	34.3	67.5	24.7	3.7	5.1
	Ah3	14–24	6.6	444	100	62.3	0.3	4.9	0.92	11.1	6.1	14.3	6.1	34.5	67.8	26.0	2.9	5.3
	Ah4	24–35	6.9	439	100	37.7	1.0	3.3	0.89	12.5	6.8	17.7	ND	38.7	77.7	27.8	2.8	5.4
	BA	35–43	7.0	412	100	39.4	2.7	3.0	0.83	18.1	6.8	15.8	ND	37.7	75.6	26.8	2.5	5.2
	C1	43–53	7.3	396	100	37.6	18.2	2.0	0.80	61.3	6.4	15.4	ND	32.8	67.2	23.6	2.0	4.8
	C2	53–68	7.4	294	100	44.3	27.4	1.7	0.85	88.5	6.2	15.2	ND	28.9	60.1	21.0	1.7	4.1
*Tuttlingen*	Oi	8–5	5.5	ND	ND	492.4	0	13.0	0.50	23.0	1.9	1.4	0.1	0.6	0.5	ND	ND	ND
*SW*	Oe	5–0	5.6	602	89	458.3	0	20.4	0.81	26.9	2.2	3.3	0.4	3.6	6.3	ND	ND	ND
	Ah1	0–11	6.8	455	95	140.1	1.7	9.3	0.90	28.3	7.0	17.9	2.8	32.7	68.3	19.1	2.9	6.5
	Ah2	11–18	7.1	424	100	83.0	9.8	6.3	0.87	36.5	7.5	18.6	2.9	36.2	74.7	21.5	2.7	7.2
	Ah3	18–25	7.1	391	100	68.7	14.7	5.3	0.78	67.9	7.2	18.9	3.5	33.5	69.8	23.8	2.5	6.6
	CA1	25–29	7.1	339	100	60.6	14.0	5.1	0.70	55.7	6.2	16.0	5.9	29.9	66.6	19.0	2.3	5.9
	CA2	29–36	7.3	327	100	51.9	25.2	4.2	0.70	90.3	6.0	15.0	5.6	27.4	62.1	18.9	2.2	5.9
	CA3	36–42	7.2	270	100	32.8	42.3	3.0	0.62	140	5.3	12.8	4.6	22.0	40.4	16.0	1.5	4.3
	CA4	42–60	7.4	183	100	25.8	51.5	2.1	0.55	184	4.8	11.3	4.1	17.6	32.7	12.9	1.3	3.9
*Bärenthal*	Oie	2–0	5.3	1017	52	324.4	0	15.6	0.79	9.9	2.2	5.6	ND	18.9	25.8	ND	ND	ND
	Ah1	0–5	4.8	362	60	119.2	0	4.7	0.72	5.3	3.4	9.0	ND	40.4	63.4	33.9	2.3	2.0
	AB	5–16	4.8	338	49	52.2	0	3.3	0.67	3.5	3.9	10.5	ND	54.0	74.2	34.8	2.3	2.2
	BA	16–36	5.3	319	67	18.1	0	1.3	0.59	4.3	4.3	10.8	ND	65.7	93.6	37.3	1.9	2.1
	Bw	36–52	6.5	316	90	11.2	0	0.79	0.50	7.1	4.6	10.3	ND	65.7	106	38.8	0.9	1.9
	CB	52–80	7.3	255	100	18.5	40.3	0.79	0.49	139	2.7	4.6	9.5	64.5	64.2	45.9	0.4	0.9
*Schänis*	Oi	2–1	5.1	ND	ND	451.6	0	11.6	0.53	24.0	2.6	4.1	0.0	4.0	3.9	ND	ND	ND
	Oe	1–0	5.8	509	97	368.3	0.0	18.5	1.23	21.8	2.8	4.9	0.0	9.7	11.4	ND	ND	ND
	Ah1	0–5	6.2	320	97	66.1	0.0	6.5	0.94	7.7	4.9	12.6	1.7	27.1	34.1	13.6	5.7	3.3
	Ah2	5–10	6.6	317	100	37.4	0.0	4.2	0.86	6.8	5.1	16.7	2.5	28.7	36.0	13.8	5.7	3.5
	Ah3	10–20	6.5	215	100	27.4	0.0	3.5	0.79	6.0	4.9	14.3	2.2	28.7	35.4	16.1	6.2	3.6
	Bw1	20–35	6.6	188	100	17.5	0.0	2.3	0.67	5.5	4.7	10.9	1.8	28.1	34.8	16.5	6.1	3.5
	Bw2	35–55	6.6	169	100	13.3	2.7	1.9	0.52	5.1	4.7	9.7	1.3	28.3	37.7	17.1	5.7	3.0
	CB	55–85	7.2	118	100	7.4	1.5	1.7	0.41	4.3	5.0	11.1	1.2	28.5	34.8	17.3	4.2	2.1
	CB	85–100	7.2	142	100	9.7	0	1.6	0.64	8.9	6.1	16.1	1.7	31.9	38.4	18.0	4.5	2.4

### Methods

We tested our hypotheses by investigating the carbonate-derived soils applying the exactly same methods of sampling, pretreatment, and analysis as used before by Lang et al. (2017) and Prietzel et al. (2016b: XANES studies) for the silicate-derived soils. We (i) characterized the carbonate-derived soils regarding contents and stocks of total P and different P forms. Moreover, we (ii) compared their P status with that of silicate-derived soils under similar climate and vegetation cover. Furthermore, we (iii) assessed the P nutrition strategy of the forest ecosystems on the carbonate sites.

#### Soil sampling and pretreatment

For all soil analyses except microbial biomass and enzyme analysis (see below), we used soil samples derived from volume-based sampling of a complete soil profile performed over an area of 0.25–0.56 m^2^, down to the consolidated bedrock or 100 cm depth, whichever was reached first. Due to the high stone content of all soils except of *BAE*, we used the ‘‘quantitative soil pit’’ (QP) approach developed by Hamburg (1984) as modified by Vadeboncoeur et al. (2012). By analyzing a soil volume with a large cross section representing a large portion of the rooting space of an adult tree, QP sampling provides a more coherent representation of the system than analysis of several small soil volumes. We established four pits at *MAN,* two pits at *TUT*, and one pit at *SCH*. Details of the QP approach and our soil sampling procedure are reported in the Supplementary Information. At *BAE*, soil material for chemical analyses was sampled from the face of a profile. Bulk density and coarse fragment data were taken from Stahr and Böcker (2014). For microbiological and enzyme analyses, we sampled Oe, Oa, and Ah material at representative locations at < 1 m distance of the QPs. All microbiological and enzyme samples were frozen immediately after sampling with solid CO_2_ (“dry ice”) and kept frozen until analysis.

#### Determination of total element contents

Total contents of P, K, Ca, Mg, K, Na, Al, Fe, Mn, and Ti in all soil samples were analyzed by digestion of fine-ground subsamples with a mixture of concentrated HClO_4_/HNO_3_/HF (Jackson 1958; Lim and Jackson 1982) and analysis by inductively coupled plasma-optical emission spectrometry ICP-OES (Varian Vista-Pro CCD).

#### Determination of different soil P forms

#### Wet-chemical determination of organic, inorganic, and plant-available P

We analyzed organic P (P_org_) by extracting sieved soil samples with 0.5 M H_2_SO_4_ before and after ignition at 550 °C (Saunders and Williams 1955). Organic P was calculated as difference of orthophosphate (oPO_4_) determined in extracts from ignited and respective non-ignited subsamples, using the malachite green colorimetric method (Ohno and Zibilske 1991). Uncertainties related to this approach are discussed in Lang et al. (2017). Plant-available P was extracted from sieved soil samples with 0.5 M NaHCO_3_ adjusted to pH 8.5 with NaOH (Olsen et al. 1954). Orthophosphate-P in the NaHCO_3_ extracts was analyzed photometrically, using the ascorbic acid method of Murphy and Riley (1962) as modified by John (1970), and total P in the extracts was analyzed by ICP-OES. The difference between oPO_4_-P and total P was assumed to be organic P.

#### P K-edge XANES spectroscopy

On fine-ground mineral soil samples, we acquired P K-edge X-ray absorption near-edge spectroscopy (XANES) spectra at beamline 8 of the Synchrotron Light Research Institute (SLRI) in Nakhon Ratchasima, Thailand (Klysubun et al. 2012; 2019). The instrument is equipped with a InSb(111) double-crystal monochromator (energy resolution ∆E/E: 3*10^–4^), which was calibrated with elemental P (E_0_ = 2145.5 eV). We recorded all spectra in fluorescence mode with a 13-element Ge detector. For each sample, depending on its P content, we acquired between two and five spectra. The spectra were deconvoluted for quantification of different soil P species by linear combination fitting (LCF) according to Werner and Prietzel (2015). For LCF, we used spectra of the reference compounds FePO_4_, AlPO_4_, hydroxy apatite, CaHPO_4_, phytic acid Na salt hydrate (IHP), Ca phytate, Fe(III) phytate, oPO_4_ as well as IHP adsorbed to boehmite, ferrihydrite, and Al-saturated montmorillonite (Prietzel et al. 2016a). All standards were diluted with fine-ground quartz to 2 mg P g^−1^ to avoid self-absorption. Phosphorus speciation shares < 5% of total P were excluded from the result list, and LCF was repeated without the respective standard. To improve LCF accuracy and precision, we used the averages of the five “best” results with the smallest R factors according to Eriksson et al. (2015). The K-edge XANES spectra of inorganic and organic P adsorbed to the same mineral are very similar (Prietzel et al. 2016a) and thus hard to quantify by LCF (Gustafsson et al. 2020). For proper identification of these P forms, we therefore combined the XANES P speciation data with the results of the wet-chemical determination of organic and inorganic P (see Supplementary Information).

#### Hedley fractionation

We analyzed sieved soil samples using the sequential extraction method of Hedley et al. (1982) as modified by Tiessen and Moir (1993). For each sample, we extracted 0.5 g soil with solutions of increasing P mobilization strength. We started with deionized water containing an anion exchange resin (Dowex 18, 20–50 mesh, Sigma-Aldrich, Taufkirchen, Germany). This was followed by extractions with 0.5 M NaHCO_3_, 0.1 M NaOH, 1 M HCl, concentrated HCl (7 M), and a final digestion with 65% HNO_3_/37% HCl (*aqua regia*). Orthophosphate concentrations in the extracts were determined according to Murphy and Riley (1962). Fractions were combined to estimate the following P pools (Niederberger et al. 2019): *Labile P:* P extractable by resin or NaHCO_3_; *Moderately labile P:* P extractable by NaOH or 1 M HCl; *Stable P*: P mobilized by HCl (conc.) or *aqua regia* digestion.

#### ^31^P NMR spectroscopy

The speciation of organic P in the Ah horizons of soils *MAN* and *TUT* was analyzed by ^31^P-NMR spectroscopy of soil NaOH-EDTA extracts. The method and key results have already been described by Wang et al. (2020). Briefly, we extracted the samples with 0.25 M NaOH plus 0.05 M Na_2_-EDTA (1:1 v/v) according to Cade-Menun (2005). Then we centrifuged the samples (1500 × g, 20 min), and split the supernatant into two equal portions. One portion was lyophilized directly (Thermo Freeze Dryer, Heto PowerDry PL6000). The second portion was dialyzed (molecular weight cutoff: 14,000; thickness: 0.041 mm; Visking, Cellulose, Roth, (Sumann et al. 1998; Amelung et al. 2001). Subsequent sample preparation for NMR spectroscopy included resolving the freeze-dried extracts in 1 ml *aqua dest.* and additionally 0.5 ml D_2_O and 10 M NaOH in order to increase and standardize the pH for optimal peak separation (Crouse et al. 2000). Then we centrifuged all samples (1500 × g, 20 min) und decanted the supernatants into NMR tubes. For spectra acquisition, we used a Varian 600 MHz spectrometer equipped with a 5 mm broadband probe tuned to the ^31^P nucleus. Other parameters were listed as: 45° pulse calibrated at 6.0 μs, 0.4 s acquisition time, 5 s total relaxation delay, 15,800 scans, proton inverse-gated decoupling, and a temperature of 293.15 K. Chemical shifts of signals were measured in parts per million (ppm) relative to 85% H_3_PO_4_. For each sample, we acquired approximately 25,000 scans. All spectra were recorded with a line broadening of 3.0 Hz. Terminology and interpretation of the spectra followed Cade-Menun (2005; 2015), Bol et al. (2006), and Vincent et al. (2013). We analyzed the spectra as described by Turner (2004).

#### Determination of microbial P

Microbial biomass P (P_mic_) was determined on selected samples of *MAN N1*, *TUT NW*, and *SCH* in triplicate using anion-exchange resin membranes by simultaneous liquid fumigation and extraction (Kouno et al. 1995) with hexanol instead of liquid chloroform (Bünemann et al. 2004). Four gram of field-moist soil were shaken together with resin and distilled water either with hexanol or without, subsequently eluting the resin with 0.1 M NaCl/HCl. The P concentration in the eluate was determined by the malachite green method (Ohno and Zibilske 1991). We determined P_mic_ according to Bünemann et al. (2016) using Eq. 1:

1 $$P_{mic} = {{(P_{fum} - P_{resin } )} \mathord{\left/ {\vphantom {{(P_{fum} - P_{resin } )} {P_{rec} }}} \right. \kern-\nulldelimiterspace} {P_{rec} }}$$

where P_fum_ and P_resin_ are the concentrations of P (in mg P kg^−1^) extracted from fumigated and non-fumigated subsamples, respectively, and P_rec_ is the fraction of the added P spike that is recovered on the resin membranes, which is calculated following Eq. 2:

2 $$P_{rec} = {{(P_{spike} - P{}_{resin })} \mathord{\left/ {\vphantom {{(P_{spike} - P{}_{resin })} {P^{*} }}} \right. \kern-\nulldelimiterspace} {P^{*} }}_{spike}$$

where P_spike_ is the P concentration measured in the P-spiked subsample and P*_spike_ is the amount of P added with the spike (both in mg P kg^−1^). In accordance with Bünemann et al. (2016) we present P_mic_ without the use of a conversion factor which could account for incomplete extraction of microbial P since this factor is method- and soil-dependent (Oberson and Joner 2005) but was not determined in our study.

#### Assessment of bacterial and fungal biomass

The relative contribution of bacterial and fungal biomass to total microbial biomass in Oe, Oa, and Ah horizons of the study soils was estimated by phospholipid fatty acid (PLFA) analysis conducted on field-moist samples. Fungal and bacterial PLFAs were determined according to Bligh and Dyer (1959) with modifications as described by White et al. (1979) and Bardgett et al. (1996). Gram-positive bacterial biomass was quantified using the fatty acids i15:0, a15:0, i16:0, and i17:0. Gram-negative bacterial biomass was quantified using the fatty acids cy17:0 and cy19:0. For total bacterial PLFAs, the sum of gram-positive and gram-negative bacterial fatty acids as well as of the fatty acid 16:1ω7 were used (Frostegård et al. 1993). For fungal biomass (accounting for saprotrophic fungi and ectomycorrhizal biomass) the fatty acid 18:2ω6 was used (Federle et al. 1986).

#### Determination of acid phosphomonoesterase and phosphodiesterase activities

Acid phosphomonoesterase (EC 3.1.3.2) activity was determined using a modified disodium phenylphosphate method. Briefly, each soil sample (field-moist, stored at –20 °C and sieved) was split into three subsamples and two controls of 1 g each. Soil suspensions were prepared with 10 ml acetate buffer (pH 5) and 5 ml 20 mM disodium phenylphosphate (EC 3279–54-7) as substrate solution; in controls, substrate solution was replaced by deionized water. All suspensions were incubated at 37 °C and continuous shaking (100 rpm) for 3 h. The release of phenol was determined colorimetrically at 614 nm (ELx808, Absorbance Microplate Reader, BioTek Instruments, Winooski, VT, USA), using 2,6-dibromquinone-chlorimide (EC 202–937-2) as coloring reagent (Hoffmann 1968, modified by Öhlinger 1996). Phosphodiesterase activity (EC 3.1.4.1) was measured using bis(*p*-nitrophenyl) phosphate (EC 223–739-2) as substrate and bis(hydroxymethyl) aminomethane as the *p*-nitrophenol color reagent according to a modified procedure of Margesin (1996). Each fresh soil sample was split into three subsamples and two controls of each 1 g. Soil suspensions were prepared with 4 ml 0.05 M Tris(THAM) buffer (pH 8.0) and 1 ml 5 mM substrate solution. In controls, substrate solution was replaced by deionized water. Soil suspensions were incubated at 37 °C for 1 h at continuous shaking (100 rpm). After incubation, 1 ml 0.5 M NaCl solution and 4 ml 0.1 M Tris(THAM) buffer (pH 12.0) were added to each subsample, whereas the controls received additionally 1 ml of the substrate solution. Soil suspensions were filtered and pipetted into 96-well microplate (PS F transparent 96 well; Greiner Bio-one, Frickenhausen, Germany). The enzyme activity was measured photometrically at 405 nm on a micro-plate reader (ELx808, BioTek Instruments, Winooski, VT, USA).

#### Isotopic exchange kinetics

For determination of isotopic exchange kinetics (IEK), we added a given amount of H_3_^33^PO_4_ to a pre-equilibrated (*i.e.* steady-state conditions for P) soil:water (100 ml:10 g) suspension and measured the decrease of radioactivity in the solution over time. At the end of the experiment (after 90 min), we determined water extractable P (*P*_*w*_) after filtration of the soil solution (0.2 µm) using the malachite green method (Ohno and Zibilske 1991). The decrease in solution concentration of the initially added ^33^P can be described by Eq. 3 (Fardeau 1993)

3 $$r_{t} /R = m \times \left( {t + m^{1/n} } \right)^{ - n} + r_{\infty } /R$$

where *r*_*t*_ and *r*_*∞*_ (MBq) are the radioactivity remaining in solution after *t* min and after an infinite time of isotopic exchange, respectively. *R* (MBq) is the initially added radioactivity, *t* (min) is the time elapsed after radioactivity addition, and *m* and *n* are soil-specific parameters calculated from a non-linear regression between *r*_*t*_/*R* and *t* after Frossard and Sinaj (1997). The *r*_*∞*_/*R* value is estimated as the ratio of water extractable P to total inorganic P (both in mg P kg^−1^). As described by Fardeau (1993), the amount of isotopically exchangeable P (*E*_*t*_, in mg P kg^−1^ soil) is calculated using Eq. 4 as described by Fardeau (1993):

4 $$E_{t} = P_{w} \times ({R \mathord{\left/ {\vphantom {R {r_{t} }}} \right. \kern-\nulldelimiterspace} {r_{t} }})$$

We calculated the following variables: *m*, *n*, P_w_ and the amounts of P isotopically exchangeable within 1 min (*E*_*1min*_, mg P kg^−1^ soil), between 1 min and 1 day (*E*_*1min–1 day*_), between 1 day and 3 months (*E*_*1day–3 months*_). Additionally, we calculated the amount of P that cannot be exchanged within 3 months (*E*_>*3 months*_) by taking the difference between total inorganic P obtained by extraction following Saunders and Williams (1955) and *E*_*3months*_.

#### Other soil variables with relevance for the soil P status

Contents of total soil carbon (C) and nitrogen (N) were determined on dried (105 °C), sieved (2 mm), and fine-ground samples using an elemental analyzer (Vario EL cube, Elementar, Hanau, Germany). On subsamples, inorganic C (carbonate) contents were determined by excess addition of 4 M HCl and quantification of the released CO_2_ using a calcimeter (Eijkelkamp, Giesbeek, The Netherlands). The pH of air-dried, sieved samples was determined in deionized water and in 1 M KCl at soil:solution ratios of 1:2.5 (w/v). Cation exchange capacity (CEC) and exchangeable cations were determined using NH_4_ acetate at pH 7 and KCl (Hendershot et al. 2008). Concentrations of extracted Ca, Mg, K, and Na were analyzed by ICP-OES (Ultima 2, Horiba Jobin–Yvon, Longjumeau, France). NH_4_^+^ in the KCl extracts was determined using an automated photometer (SANplus, Skalar Analytical, Breda, The Netherlands). The difference between the CEC and the sum of Ca, Mg, K, and Na is an estimate of H^+^ and Al^3+^ occupation of the CEC. We applied the hot dithionite–citrate–bicarbonate extraction method of Mehra and Jackson (1960) to estimate total pedogenic Fe oxyhydroxides (Fe_d_). Extraction with NH_4_ oxalate at pH 3.0 and 2 h shaking in the dark (Schwertmann 1964) was carried out to estimate Al and Fe in organic complexes and short range-ordered (SRO) minerals (Al_o_; Fe_o_). Concentrations of extracted Al and Fe were analyzed by ICP-OES. Soil microbial biomass C and N (C_mic_; N_mic_) were determined using the chloroform fumigation extraction (CFE) method (Brookes et al. 1985; Vance et al. 1987). Non-fumigated, moist soil (7 g) was extracted with 30 ml 0.05 M K_2_SO_4_ for 1 h (Bruulsema and Duxbury 1996) by overhead shaking (40 rev min^−1^). A similar amount of soil was fumigated with ethanol-free chloroform and extracted in the same way. The fumigation was carried out in desiccators at 20 °C for 24 h. The organic C content of the extracts was measured using a CN analyzer (2100 S, Analytik Jena). Microbial biomass C and N were calculated by dividing the microbial C or N flush (E_C_; E_N_); the difference between extracted C or N from fumigated and non-fumigated soil samples with k_EC_ or k_EN_ factor of 0.45 (Wu et al. 1990).

#### Conversion of element and P species contents into soil stocks

Contents of various soil constituents in different soil horizons were converted into stocks by multiplying the content data with the surface area-standardized soil mass of a given horizon as retrieved by the QP approach and in the case of *BAE* with the data reported by Stahr and Böcker (2014). The stock values of the various horizons comprising a soil profile were summed up to yield the total soil stock.

#### Assessment of ecosystem P nutrition strategy

The type of ecosystem P nutrition (P-acquiring *vs.* P-recycling; Lang et al. 2016; 2017) was assessed for all eight carbonate soil profiles. For each profile, we calculated the values of the three indicators for P acquisition and of the four indicators for P recycling (Table 3) as described by Lang et al. (2017). Due to limited data availability, N2 and N3 could only be calculated for four carbonate sites, and N6 only for two sites. To enable comparison of the different indicators among the carbonate soils and also between carbonate and silicate soils, we normalized the indicator values obtained for each carbonate profile as in the study of Lang et al. (2017), using Eq. 5.

5 $$N_{ai} = \frac{{I_{ai} }}{{I_{am} }}$$

**Table 3:** Indicators for ecosystem P-acquisition and P-recycling, method of calculation of indicator values and justification of indicator (from Lang et al. 2017)

Variable	Calculation of indicator values	Assumed underlying process
P acquiring indicators		
N1: P-enrichment in topsoil	P stock in the upper 50% of soil fine earth mass divided by total soil P stock (up to 1 m)	Spatial redistribution induced by the P pumping of trees in the long term: root uptake of P in the subsoil, P deposition with litter at the topsoil and adsorption after mineralization
N2: Proportion of nonstable P in profile	Stock (up to 1 m) of non-stable P. (i.e., sum of Hedley P minus P_HCl conc_ and P_residual_) relative to total Hedley P	Chemical redistribution due to biological mobilization of P from primary minerals. Nutrient demand had been discussed as the reason for root induced weathering
N3: Phosphate exchangeability between 1 min and 1 day	Concentration of isotopically exchangeable P between 1 min and 1 day of topsoil horizons as described in methods chapter	P exchangeability based on physicochemical processes; indicator for P availability
P recycling indicators		
N4: Accumulation of P in forest floor	P stock in the forest floor related to total P stock (up to 1 m mineral soil depth)	Forest floor pathways as short cut for plant P uptake without passing of P through the fixing mineral soil
N5: Concentration of fine-root biomass in forest floor	Total fine root biomass in forest floor and upper 0–5 cm mineral soil in relation to total fine root biomass (up to 1 m mineral soil depth)	Peak concentrations of fine roots in the forest floor have been assumed to favor tight P cycling in acid temperate forest ecosystems. Results are clearer when the 0–5 cm increment of the mineral soil is added
N6: Enrichment of diester-P	Diester-P/monoester-P ratio in the topsoil horizon as calculated from NMR spectra	Increased proportions of diester P were observed in acid soils and explained by changes in enzyme activity, and decreased accessibility of diester P for microbial decay due to accumulation within large organic molecules
N7: Mean residence time of forest floor SOM	Forest floor mass related to the mass of annual litter fall	Limited decay of soil organic matter enhances tight P recycling by providing forest floor P-pathways for tree nutrition

where *N* represents the normalized indicator value, the index *a* the indicator addressed, the index *i* the study site, and *I* the indicator value. The index *m* represents the P-richest site on silicate bedrock (*Bad Brückenau*; *BBR*), characterized as P-acquiring ecosystem, and the P-poorest site on silicate parent material (*Lüss; LUE*) characterized as P-recycling ecosystem. For an overall estimate of the P nutrition strategy of each carbonate and silicate site, we first calculated the arithmetic mean of the three P acquisition indicator values obtained for the individual sites. Then, we referenced the average P acquisition indicator to the range of average P acquisition indicators of the silicate sites*.* Their respective average P acquisition indicators were defined as 1 (*BBR* = silicate site with maximum P supply; P-acquiring) and 0 (*LUE* = silicate site with minimum P supply; P recycling; Lang et al. 2017). Accordingly, we calculated the arithmetic mean of the four P recycling indicator values for each site, and referenced it to the range of average P recycling indicators of silicate sites, with that of *BBR* defined as 0 and that of *LUE* defined as 1. Finally, an ecosystem P nutrition index *(ENI*_*P*_*)* was calculated for each site by subtracting the referenced mean P recycling indicator from the referenced mean P acquisition indicator of each site. Thus, the P nutrition strategy of each site was related to a scale ranging from *ENI*_*P*_ =  +1 (acquiring endmember *BBR* of silicate sites) to –1 (recycling endmember *LUE* of silicate sites). A positive *ENI*_*P*_ indicates predominance of P-acquiring over P-recycling, whereas a negative *ENI*_*P*_ indicates predominance of P-recycling over P-acquiring.

## Results

### Total soil P contents and stocks

**Fig. 1:**
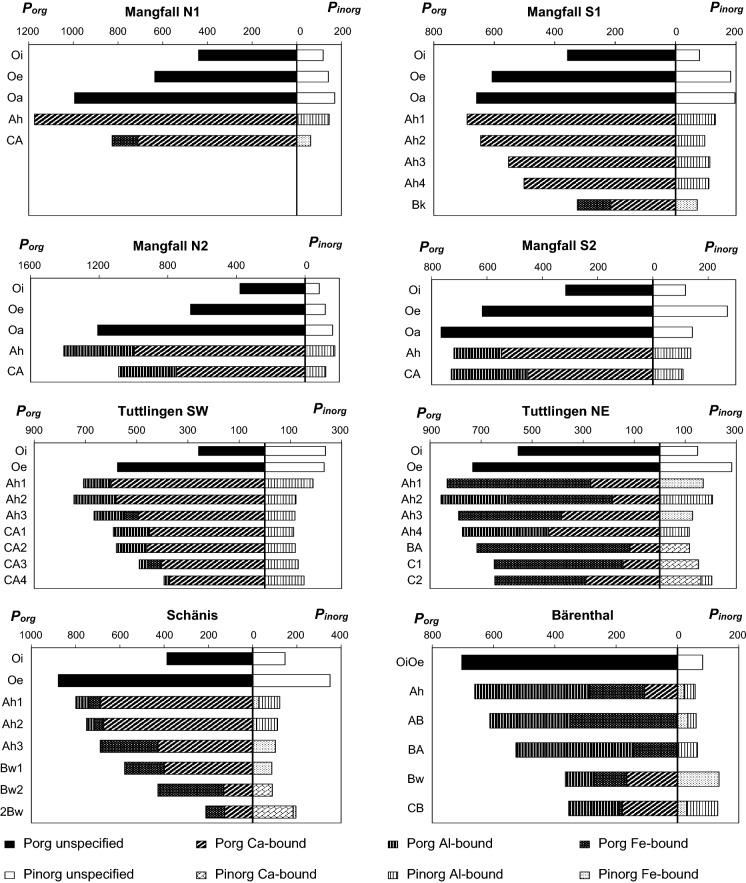
P speciation of the soils at sites *Mangfallgebirge*, *Tuttlingen*, *Bärenthal,* and *Schänis*. Presented are contents of different organic P species (left from vertical zero axis) and inorganic P species (right from vertical zero axis) in mg P kg^−1^ soil

**Fig. 2:**
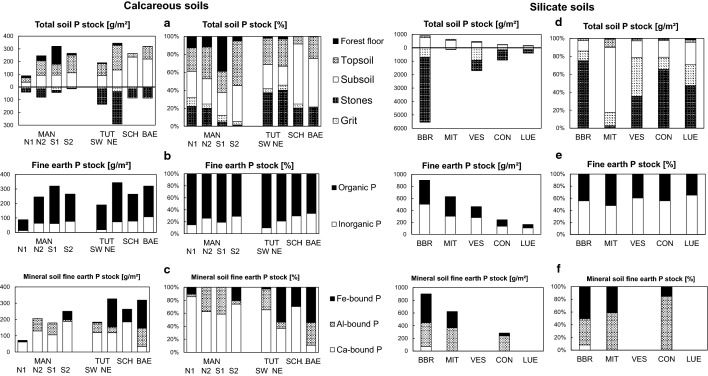
Comparison of soil P stock speciation at temperate beech forest sites with carbonate **a-c** and silicate **d-f** parent material. Absolute and relative contribution of **a,d** different soil compartments, **b,e** organic and inorganic fine earth P, and **c,f** Ca-bound, Al-bound, and Fe-bound mineral soil fine earth P. Sites with dolostone parent material: *Mangfallgebirge* (MAN; N1, N2: North slope, S1, S2: South slope). Sites with limestone parent material: *Tuttlingen* (TUT; SW: Southwest slope; NE: Northeast slope), *Schänis* (SCH), *Bärenthal* (BAE). Sites with silicate parent material (Data from Prietzel et al. (2016a, b, c) and Lang et al. (2017)): *Bad Brückenau* (BBR), *Mitterfels* (MIT), *Vessertal* (VES), *Conventwald* (CON), *Lüss* (LUE)

At all sites, total P contents (Table 2; Fig. 1) in the forest floor increased with depth and degree of SOM decomposition in the sequence Oi–Oe–Oa horizon, whereas those in the mineral soil decreased with depth. Total soil fine earth P stocks ranged between 90 and 350 g m^−2^ (Fig. 2a). The relative contribution of P bound in coarse fragments to the total P in the profiles ranged from 5% at *MAN S1* to 42% at *TUT SW*. The forest floor comprised up to 44% of total soil (fine earth) P in the P-poor *MAN* profiles, whereas its contribution was small (< 5%) in the other soils. The contribution of the topsoil (Ah horizons) to total soil (fine earth) P ranged between 11 and 56%, while the subsoil (B, C horizons) contributed between 29 and 89% to total soil (fine earth) P. In the P-rich profiles *SCH* and *BAE* with advanced pedogenesis and thick B horizons, subsoil P strongly dominated the soil P pool.

### Contents and stocks of different soil P forms

#### Organic and inorganic P

In all soil horizons organic P (P_org_) dominated over inorganic P (P_inorg_), and in almost all horizons P_org_ comprised > 70% of total P (Fig. 1). Organic P contents generally decreased with increasing mineral soil depth. In contrast, P_inorg_ contents often showed a secondary maximum in the deep subsoil, but were always smaller than P_org_ contents. Organic soil P stocks (Fig. 2b) ranged between 75 and 271 g m^−2^ and comprised between 66–70% (in Cambisols *SCH* and *BAE*) and 90% (Rendzic Leptosol *TUT SW*), on average 77% of total soil (fine earth) P. Inorganic soil P stocks ranged between 13 and 108 g m^−2^ and comprised between 10% and 30–34% (*SCH* and *BAE*), on average 23%, of total soil P (Fig. 2b).

#### P speciation in mineral soil horizons assessed by P K-edge XANES spectroscopy

According to P K-edge XANES (Fig. 1, Fig. 2c; Table S2), P_org_ in the mineral soil of the dolostone-derived soils as well as in *TUT SW* and *SCH* was predominantly Ca-bound. On average, about 60% of mineral soil fine earth P was Ca-bound; Fe-bound P and Al-bound P each constituted about 20%. A difference in soil P speciation can be noticed between soils without B horizons (*MAN N1, N2, S2; TUT SW*) and soils with B horizons (*MAN S1, SCH,* particularly *BAE*) or impure carbonate bedrock (marl limestone at *TUT NE*). The latter soils had larger Fe-bound P stocks, which constituted the majority of fine earth P at *BAE* and *TUT NE.*

#### *NaHCO*_*3*_*-extractable P*

**Fig. 3:**
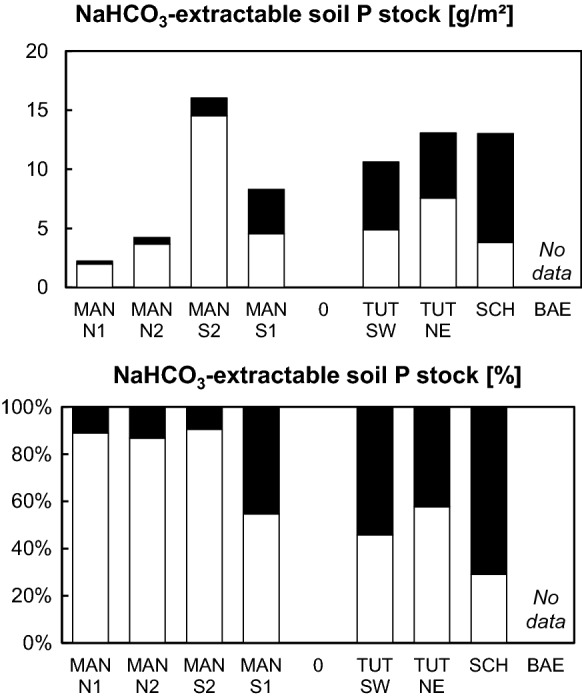
Plant-available soil P stocks (NaHCO_3_-extractable P) at temperate beech forest sites with carbonate parent material. Shown are absolute and relative contributions of organic P (black bars) and inorganic P (white bars). For a detailed description of sites, please read caption of Fig. 2

Contents of NaHCO_3_-extractable P (Table S2), which is an estimate for plant-available P, decreased in the mineral soil with depth. Stocks of NaHCO_3_-extractable oPO_4_ (Fig. 3) ranged between 2 (*MAN N1*) and 15 g m^−2^ (*MAN S2*), those of NaHCO_3_-extractable P_org_ ranged between 0.2 g m^−2^ (*MAN N1*) and 9 g m^−2^ (*SCH*). NaHCO_3_-extractable P was mostly oPO_4_ at *MAN*, to about equal shares oPO_4_ and P_org_ at *TUT*, and mostly P_org_ at *SCH*.

#### Hedley P fractions

**Fig. 4:**
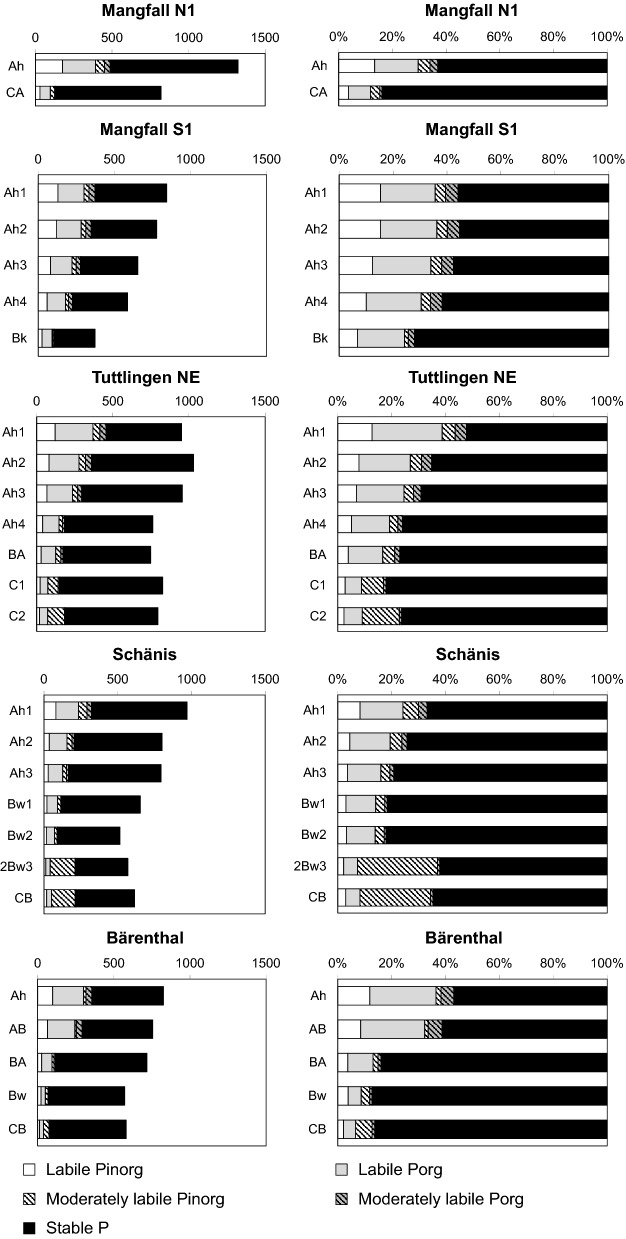
Contents (left panels; mg P kg^−1^) and relative contribution (right panels) of different Hedley P fractions (labile P: resin + NaHCO_3_-extractable P; moderately labile P: NaOH-extractable + 1 M HCl-extractable P; stable P: 7 M HCl-extractable + *aqua regia*-extractable P) in different mineral soil horizons of profiles *Mangfallgebirge* N1 and S1, *Tuttlingen* NE, *Schänis,* and *Bärenthal*

**Fig. 5:**
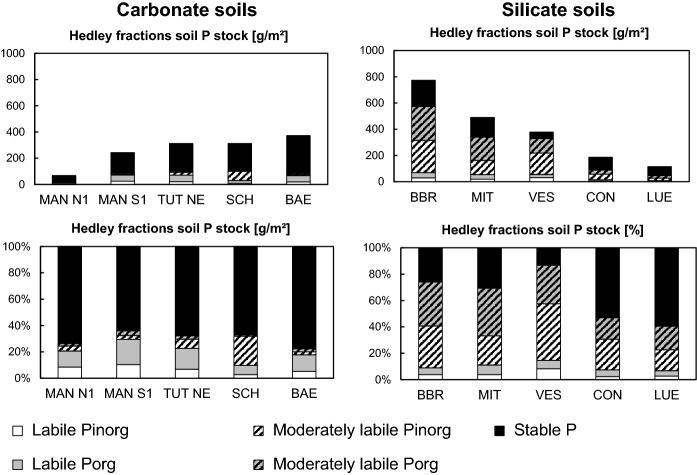
Comparison of mineral soil stocks of different Hedley P fractions at temperate beech forest sites with carbonate (left panels) and silicate parent material (right panels; data from Lang et al. (2017). Shown are absolute and relative contributions of different Hedley P fractions in the investigated profiles

In almost all mineral soil samples, the majority of soil P could only be mobilized by treatment with concentrated HCl or *aqua regia* (Fig. 4; Table S3), and thus was in the stable fraction according to Niederberger et al. (2019). The contribution of stable P to total P increased with soil depth, whereas the contribution of labile P (resin-P, NaHCO_3_-extractable P fractions) decreased. Except for the subsoil at *SCH*, < 10% of soil P was moderately labile. Mineral soil stocks of stable P increased with soil development and were larger in limestone-derived than in dolostone-derived soils (Fig. 5). The contribution of labile P to total P decreased with progressing pedogenesis.

#### *P speciation in Ah horizons by *^*31*^*P NMR spectroscopy*

**Fig. 6:**
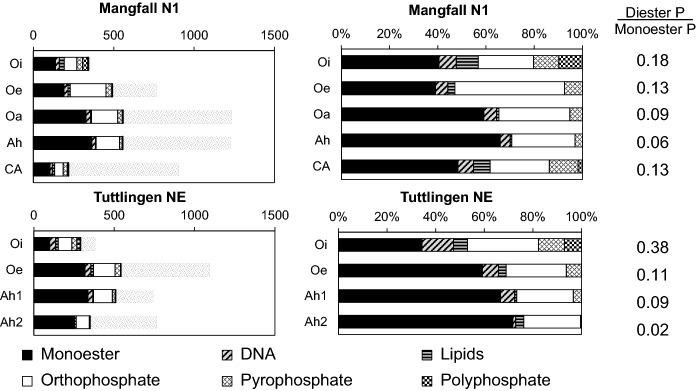
Phosphorus forms quantified by ^31^P-NMR spectroscopy of soil NaOH-EDTA extracts from O layer and mineral topsoil horizons of profiles *Mangfallgebirge N1* and *Tuttlingen NE* (Wang et al. 2020). Left: Contents in mg P kg^−1^; bars in shaded gray represent total P in respective horizon. Right: Contribution of different P forms to total extractable P

Contents of NaOH-EDTA-extractable P at *MAN N1* and *TUT NE* were about 300 (L horizons), 500 (O, Ah), and 200 (CAh) mg g^−1^ soil (Fig. 6; Wang et al. 2020). Extraction recoveries as related to total P contents of the respective horizons (shaded bars in Fig. 6) decreased with soil depth from 80–100% in L layers to 50–60% in O and Ah and 25% in CAh horizons. With 35–70% of extractable P, monoester P was the dominating P form. Its contribution to total extractable P increased with depth. Between 1 and 11% of extractable P was phosphodiester P bound in DNA, and between 1 and 9% was lipid phosphodiester P. Orthophosphate constituted about 25% of extractable P, and about 80% of extractable P_inorg_. Between 4 and 12% of extractable P was pyrophosphate-P. Polyphosphate was only present in Oi layers (7–10% of extractable P).

#### Microbial bound P and enzyme activities

**Fig. 7:**
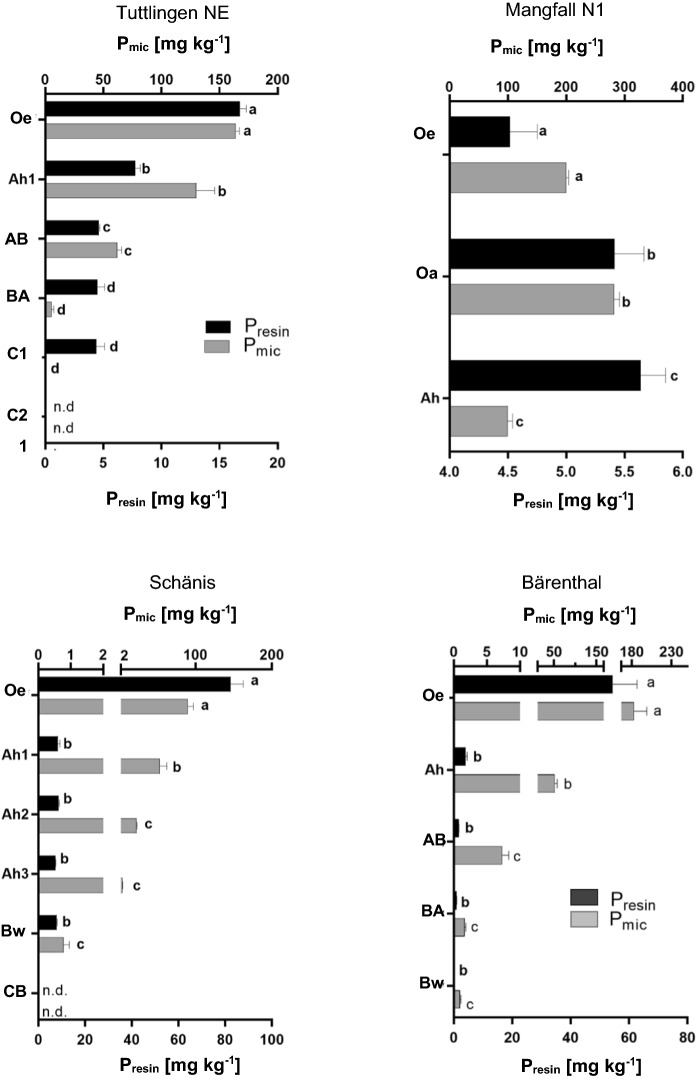
Soil microbial biomass phosphorus (P_mic_) and extractable P (P_resin_) in profiles *Mangfallgebirge N1, Tuttlingen NE, Schänis,* and *Bärenthal*. Significant differences (p < 0.05) between horizons are denoted with lower-case letters. Values below detection limit are denoted with n.d

Contents of microbial P, C, and N (P_mic_, C_mic_, N_mic_) in all profiles significantly decreased with depth (Fig. 7; Figure S2, S3). In the Ah horizons, P_mic_ contents increased in the sequence *SCH* < *MAN N1* < *TUT NE*. Mass ratios of C_mic_/P_mic_ decreased in the same order (Table 4). Phosphomonoesterase and phosphodiesterase activities also decreased with soil depth and on average were eight times (phosphomonoesterase) and three times (phosphodiesterase) larger in O than Ah horizons (Table 5). A larger decrease in phosphomonoesterase than phosphodi-esterase activities resulted in smaller phosphomonoesterase/phosphodiesterase ratios in A horizons than O layers.

**Table 4:** Contents of microbial C (C_mic_), N (N_mic_), and P (P_mic_), C_mic_/P_mic_ and C_mic_/N_mic_ mass ratios as well as mass ratios of organic C (C_org_) over organic P (P_org_) and organic N (N_org_) in the Ah1 horizons of the carbonate profiles *Mangfallgebirge N1*, *Tuttlingen NE*, and *Schänis*

Site	Method	C_*mic*_ (µg g^−1^ )	N_*mic*_ (µg g^−1^ )	P_*mic*_ (µg g^−1^ )	P_resin_ (µg g^−1^ )	C_mic_/P_mic_ (g g^−1^ )	C_mic_/N_mic_ (g g^−1^ )	C_org_/P_org_ soil (g g^−1^ )	C_org_/N_org_ soil (g g^−1^ )	Pmic/Cmic P_org_/C_org_	Nmic/Cmic N_org_/C_org_
										*(Factor of enrichment* *in soil microorganisms* *relative to SOM)*	
*Carbonate parent material*											
*Mangfallgebirge N1*	Resin	2487	440	76	6	33	5.7	208	16.4	6.3	2.7
*Tuttlingen NE*	Resin	1571	210	81	5	19	7.5	124	14.3	6.5	2.0
*Schänis*	Resin	2955	570	58	8	51	5.2	83	10.2	1.6	2.0

**Table 5:** Activities of phosphomonoesterase [MONO] and phosphodiesterase [DI] as well as phosphomono-esterase/phosphodiesterase ratios in Oe, Oa, and Ah horizons of the profiles on carbonate parent material (mean ± standard deviation, n = 3)

	Phosphomonoesterase [MONO] (pH 5.0)	Phosphodiesterase [DI] (pH 8.0)	MONO/DI
	*mg Phenol* *g*^*−1*^* soil 3 h*^*−1*^	*mg p-Nitrophenol* *g*^*−1*^* soil 1 h*^*−1*^	*Ratio*
Oe horizons			
*Mangfallgebirge N1*	124.3 ± 14.3	2.2 ± 0.2	57
*Mangfallgebirge S1*	37.7 ± 15.1	1.6 ± 0.1	23
*Tuttlingen SW*	58.8 ± 2.9	1.3 ± 0.0	45
*Tuttlingen NE*	28.8 ± 2.7	1.2 ± 0.3	24
*Bärenthal*	58.6 ± 3.3	1.3 ± 0.1	46
*Schänis*	48.0 ± 3.1	1.5 ± 0.1	33
Oa horizons			
*Mangfallgebirge N1*	53.7 ± 0.9	0.76 ± 0.03	71
*Mangfallgebirge S1*	30.5 ± 0.04	1.3 ± 0.0	24
Ah1 horizons			
*Mangfallgebirge N1*	8.5 ± 0.2	0.44 ± 0.00	19
*Mangfallgebirge S1*	8.9 ± 0.1	0.61 ± 0.01	15
*Tuttlingen SW*	6.5 ± 0.1	0.52 ± 0.00	12
*Tuttlingen NE*	5.9 ± 0.2	0.35 ± 0.01	17
*Bärenthal*	7.8 ± 0.0	0.24 ± 0.03	33
*Schänis*	4.2 ± 0.0	0.21 ± 0.01	15

#### Isotopic exchange kinetics

Water-extractable P_inorg_ contents (P_w_) at *MAN S1* and *S2, TUT NE, BAE,* and *SCH* ranged from 0.003 to 2.97 µg P g^−1^ soil and decreased with increasing depth (Table 6). The fitting parameter *m,* accounting for immediate physicochemical reactions, followed the same trends as P_w_ in *TUT NE* and *SCH*. The fitting parameter *n*, accounting for slow physicochemical reactions, was rather constant over depth at all four sites. The amount of P that was isotopically exchangeable between 1 day and 3 months (E_1day-3 months_) decreased with increasing depth at all four sites. Profiles *MAN S1* and *S2* had largest amounts of isotopically exchangeable P, and *TUT NE* (Ah) and *BAE* (Bw) had largest amounts of non-isotopically exchangeable P (E_>3 months_).

**Table 6:** Results of isotopic exchange analyses for the study sites *Mangfallgebirge*, *Tuttlingen, Schänis*, and *Bärenthal* (mean ± standard deviation, n = 3). Water extractable P (P_w_) and total inorganic P (P_i_). Fitting parameters (*m* and *n*) describing the decrease of radioactivity in the solution with time, amount of P isotopically exchangeable within 1 min (E_1min_), between 1 min and 1 day (E_1min-1 day_), between 1 day and 3 months (E_1day-3 months_), and amount of P not exchangeable within 3 months (E_>3 months_)

	P_w_ (µg P g^−1^ soil)	P_i_ (µg P g^−1^ soil)	m	N	E_1min_ ( µg P g^−1^ soil)	E_1min-1 day_ ( µg P g^−1^ soil)	E_1day-3 months_ ( µg P g^−1^ soil)	E_> 3 months_ ( µg P g^−1^ soil)
*Mangfallgebirge S1*								
Ah1	2.97 ± 0.86	134.6	0.51 ± 0.18	0.59 ± 0.03	7.5 ± 4.5	93.4 ± 11.6	30.4 ± 14.1	3.3 ± 1.9
Bk	0.19 ± 0.21	48.2	0.03 ± 0.01	0.59 ± 0.10	5.2 ± 4.5	33.8 ± 4.7	8.0 ± 7.0	1.2 ± 1.4
*Mangfallgebirge S2*								
Ah	0.66 ± 0.29	138.9	0.17 ± 0.06	0.61 ± 0.07	4.4 ± 2.6	92.3 ± 14.5	37.9 ± 13.2	4.3 ± 3.3
AC	0.48 ± 0.13	104.9	0.23 ± 0.07	0.40 ± 0.87	2.2 ± 0.9	27.2 ± 15.7	39.0 ± 10.8	36.4 ± 24.8
*Tuttlingen NE*								
Ah1	2.42 ± 0.22	174.8	0.35 ± 0.05	0.33 ± 0.01	6.8 ± 0.9	45.7 ± 5.7	61.0 ± 1.6	61.3 ± 8.1
BA	0.82 ± 0.13	103.8	0.19 ± 0.01	0.37 ± 0.02	4.1 ± 0.7	35.0 ± 2.9	39.9 ± 1.7	24.9 ± 3.4
C1	0.77 ± 0.03	141.9	0.16 ± 0.01	0.35 ± 0.00	4.7 ± 0.2	37.6 ± 1.1	52.5 ± 0.5	47.1 ± 1.6
*Schänis*								
Ah1	1.13 ± 0.21	140.8	0.36 ± 0.13	0.43 ± 0.03	3.3 ± 0.7	44.0 ± 9.8	60.5 ± 2.9	33.0 ± 12.1
Ah2	0.13 ± 0.07	103.0	0.14 ± 0.05	0.46 ± 0.07	0.9 ± 0.4	20.4 ± 11.9	42.8 ± 11.4	38.9 ± 23.5
Bw1	0.09 ± 0.01	77.7	0.05 ± 0.01	0.42 ± 0.03	1.8 ± 0.2	24.3 ± 2.8	33.7 ± 1.7	18.0 ± 4.0
*Bärenthal*								
Ah	1.14 ± 0.15	54.0	0.35 ± 0.02	0.40 ± 0.04	3.2 ± 0.4	25.1 ± 4.3	18.3 ± 1.2	7.5 ± 3.5
BA	0.05 ± 0.03	48.0	0.02 ± 0.00	0.40 ± 0.03	2.3 ± 1.3	19.6 ± 7.2	17.0 ± 3.5	9.0 ± 5.1
Bw	0.003 ± 0.000	92.0	0.01 ± 0.00	0.32 ± 0.03	0.3 ± 0.0	2.7 ± 0.4	8.5 ± 2.5	80.5 ± 2.8

### Ecosystem P nutrition indicators and ecosystem P nutrition strategies

**Fig. 8:**
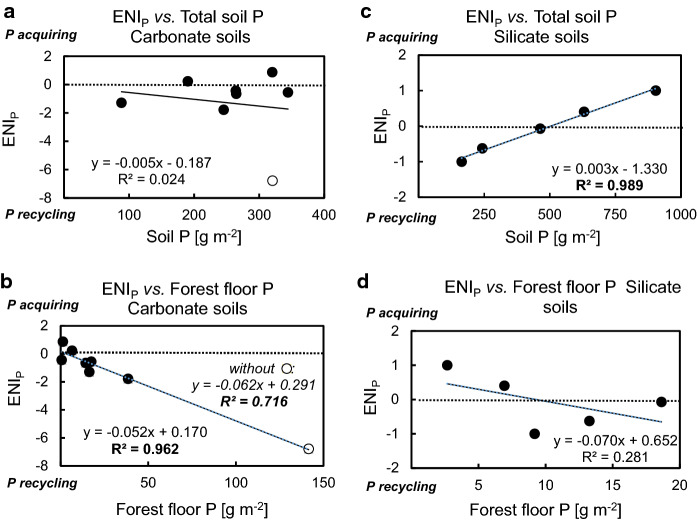
Linear regression of the Ecosystem P Nutrition Index (*ENI*_*P*_) *vs.* stocks of **a,c** total soil P, and **b,d** forest floor P in the carbonate soils **a, b** and in the silicate soils investigated by Lang et al. (2017)

The seven indicators for P acquisition (N1–N3) and P recycling (N4–N7) calculated according to Lang et al. (2017) are presented in Table 7. For three of the four *MAN* profiles, the P recycling indicators N4 and N7 ranged between 1.3 and 8.5 and thus markedly exceeded the range of the silicate soils. Consequently, negative *ENI*_*P*_ values indicated a dominance of P recycling over P acquisition at all dolostone sites (Table 7). Except for Cambisol *MAN S1*, *ENI*_*P*_ values at the dolostone sites were < –1. This indicates pronounced P recycling, exceeding even that of the most P-recycling silicate site *LUE*. Among the carbonate sites, *ENI*_*P*_ was highly negatively correlated with forest floor P (and SOC) stocks, but not with total soil P stocks (Fig. 8). The limestone sites showed a change from predominating recycling to acquiring P nutrition with progressing pedogenesis.

**Table 7:** Ecosystem P acquiring and P recycling indicators, Ecosystem P Nutrition Index *ENI*_*P*_, and phosphorus ecosystem nutrition strategy of Central European temperate beech forests on sites with different parent material. N1-N7 refer to the ecosystem P acquiring and P recycling indicators presented in Table 3. ND: Not determined. *Normalization to the interval *Bad Brückenau – Lüss* as described in Sect. Assessment of ecosystem P nutrition strategy. Dolostone and Limestone soils are ordered according to their stage of pedogenesis

Site	Acquiring indicators			Recycling indicators				Acquiring indicators		Recycling indicators		Acquiring indicators	Recycling indicators		*ENI*_*P*_	Phosphorus ecosystem nutrition strategy
	N1	N2	N3	N4	N5	N6	N7		Mean N1-N3	Mean N4-N7	Mean normalized*			Mean normalized*		
*Dolostone parent material*																
Mangfallgebirge N1	0.75	0.36	ND	3.33	1.25	0.09	1.27		0.55	1.48	0.18		1.58		−1.4	Recycling
Mangfallgebirge N2	0.76	ND	ND	2.80	1.46	ND	2.49		0.76	2.25	0.56		2.51		−2.0	Recycling
Mangfallgebirge S2	0.45	ND	0.28	7.89	1.26	ND	8.52		0.36	5.89	−0.17		6.90		−7.1	Recycling
Mangfallgebirge S1	0.93	0.49	0.31	0.96	0.74	ND	0.94		0.57	0.88	0.22		0.86		−0.6	Recycling >> Acquiring
*Limestone parent material*																
Tuttlingen SW	0.86	ND	ND	0.61	0.94	ND	0.33		0.86	0.63	0.75		0.55		0.2	Acquiring = Recycling
Tuttlingen NE	0.81	0.44	0.09	0.91	0.58	0.14	0.71		0.45	0.59	−0.01		0.50		−0.5	Recycling > Acquiring
Schänis	0.84	0.44	0.09	0.04	1.42	ND	0.03		0.46	0.50	0.01		0.39		−0.4	Recycling > Acquiring
Bärenthal	0.87	ND	ND	0.07	ND	ND	0.08		0.87	0.07	0.77		-0.12		0.9	Acquiring
*Silicate parent material*																
Bad Brückenau	**1.00**	**1.00**	**1.00**	0.05	0.31	0.20	0.13		**1.00**	0.17	**1.00**		**0.00**		**1.0**	Acquiring
Mitterfels	0.86	0.94	0.63	0.20	0.55	0.33	0.36		0.81	0.36	0.65		0.23		0.4	Acquiring > Recycling
Vessertal	0.80	1.17	0.20	0.72	0.85	0.33	ND		0.72	0.63	0.49		0.56		−0.1	Acquiring = Recycling
Conventwald	0.92	0.63	0.24	0.97	1.09	0.64	0.91		0.60	0.90	0.26		0.88		−0.6	Recycling >> Acquiring
Lüss	0.81	0.55	0.01	**1.00**	**1.00**	**1.00**	**1.00**		0.45	**1.00**	**0.00**		**1.00**		−**1.0**	Recycling

## Discussion

Many analytical methods applied in our study (*e.g.* NMR and XANES spectroscopy, Hedley P fractionation, determination of isotopic exchange kinetics) are costly and/or time demanding, preventing the analysis of replicate profiles at each study site. Therefore, our paper unfortunately does not allow for statistical analysis of soil P status differences among the various sites. Nevertheless, we think that our paper presents a lot of novel important information on the P status of soils with carbonate parent material, regarding effects of pedogenesis, bedrock carbonate purity, and differences to soils with silicate parent materials.

### Changes of P stock, P speciation, and ecosystem P nutrition in soils on carbonate bedrock with progressing pedogenesis

**Fig. 9:**
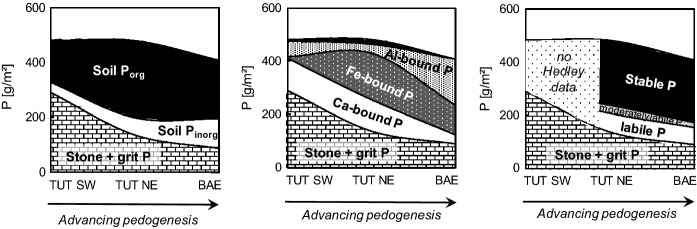
Stock changes of **a** organic and inorganic P, **b** different P forms in the mineral soil, and **c** different Hedley P fractions during pedogenesis of soils on limestone in the Swabian Alb (Tuttlingen region). TUT SW: Profile *Tuttlingen SW* (Rendzic Leptosol, age < 12,000 years). TUT NE: Profile *Tuttlingen NE*, (Rendzic Leptosol with BA horizon, age < 12,000 years. BAE: Profile *Bärenthal* (Cambisol, age > 2.5 million years). P stock bound in stone and grit of TUT NE based on a bedrock P content of 150 mg P kg^−1^

Soils *TUT SW* (shallow Rendzic Leptosol), *TUT NE* (Rendzic Leptosol with more advanced pedogenesis and a BA horizon), and *BAE* (Cambisol with thick B horizon) are located within 16 km distance from each other. They have similar parent material, climate, and forest vegetation (Table 1), but represent a series of progressing pedogenesis. Whereas the two Leptosols have developed after the last Pleistocene glaciation, and their age is < 12,000 years, Cambisol *BAE* is pre-Pleistocene and has an age of at least 2.5 Ma (Stahr and Böcker 2014). This sequence provides novel information on changes in P stock and P speciation in carbonate soils with pedogenesis. In contrast to the chronosequences on silicate parent material studied by Walker and Syers (1976), the limestone soils showed increasing fine earth P stocks (Fig. 9) with increasing soil age and progressing pedogenesis (shallow Rendzic Leptosol Cambisol). The increase in fine earth P stock was mostly caused by an increase in soil depth, formation of subsoil horizons, and fine earth (*i.e.* insoluble limestone dissolution residue + SOM) accumulation (Fig. 1). Stocks of P bound in stones and grit within the profile were decreasing faster with progressing pedogenesis than fine earth P stocks were increasing, indicating overall ecosystem P losses during pedogenesis also on carbonate sites, as shown before for silicate sites (Walker and Syers 1976; Lajtha and Schlesinger 1988; Crews et al. 1995; Chen et al. 2015). In line with the concept of Walker and Syers (1976), stocks of lithogenic Ca-bound P and the relative contribution of Ca-bound P to total soil P decreased with progressing pedogenesis (Fig. 9b). Yet, in contrast to their study, where Ca-bound P was completely lost in glacier forefield moraines after 22,000 years of soil formation under cool temperate climate, at *BAE* even after > 2.5 Ma of pedogenesis under different (humid, cold-arid, tropical) climate regimes, limestone rock fragments and Ca-bound P were still present in the Bw horizons at 50 cm depth (Fig. 1; Table 1). We assume that despite its plateau position with an inclination of only 2% *BAE* has lost a considerable portion of its pre-Pleistocene topsoil by solifluction during the Pleistocene. Forest vegetation colonizing the site in the early Holocene therefore could access and mine the underlying limestone rock, which was present at a depth < 60 cm, well within the rooting zone of forest trees, for P. At present, 15% of total P in the Ah horizon of *BAE* is Ca-bound P_org_ (Ca-IHP), probably indicating steady combined input of Ca and P with litter into the acidified topsoil (Clarholm and Skyllberg 2013).

Advancing limestone weathering, pedogenesis, and topsoil acidification in our chronosequence resulted in a decrease of Ca-bound soil P by dissolution of inorganic and organic Ca phosphates as well as accumulation of Al- and Fe-rich limestone dissolution residue, including Al and Fe oxyhydroxides. Stocks of Fe-bound P and their contribution to total soil P in our limestone-derived soils reached a maximum at intermediate stages of pedogenesis. According to the XANES results, in the old Cambisol *BAE* Al-bound P dominated over Fe-bound P, indicating that ultimately gibbsite and kaolinite were more important for soil P retention and storage than goethite and hematite. Yet, the majority of soil P in *BAE* was organic (Fig. 9a), and P K-edge XANES may have erroneously identified a considerable portion of P_org_ bound to Fe oxyhydroxides as Al-bound P (Prietzel and Klysubun 2018). Combination of the information retrieved by wet-chemical digestion, XANES, and Hedley fractionation (Fig. 9a-c) indicated that most of the P termed “occluded P” by Walker and Syers (1976), and “stable P” by Hedley et al. (1982) was Al- or Fe-bound P_org_. The latter was most likely occluded in, strongly adsorbed to, and/or co-precipitated with Al and Fe oxyhydroxides. Overall, pedogenesis in limestone soils has resulted in a long-term change from recycling to acquiring ecosystem P nutrition (Table 7), suggesting that the small (moderately) labile P stock (84 g m^−2^) in the *BAE* profile is a sufficiently large pool of ecosystem-available P for an acquiring P nutrition strategy of the beech forest at *BAE*. In summary, these results indicate that, in contrast our hypothesis (1), the concept of Walker and Syers (1976) is only ***partially valid*** for soils derived from carbonate parent material (*e.g.* soil P speciation change from Ca-bound P to Al- and Fe-bound P forms with progressing pedogenesis), and must be refuted in many aspects (decrease of total soil P, inorganic P, and labile, plant-available P stocks with progressing pedogenesis).

### Carbonate rock purity as key factor affecting soil P status and ecosystem P nutrition

Carbonate parent materials exist with different purity, *i.e.* in addition to the dominating (Ca, Mg, [Fe, Mn]) carbonates, other elements like Al, K, Na, Ca, Mg, Fe (in accessory silicate minerals) or Fe, Al, Mn (in accessory oxyhydroxide minerals) may be admixed to or co-precipitated. A well-known example is the increasing share of silicate in the sequence limestone – marl limestone – marl – marl mudstone (Blatt and Tracy 1996). Moreover, P contents of carbonate parent materials vary strongly on the global scale (Porder and Ramanchandran 2013), but also on regional and local scales (Table S1). Thus, soils formed from carbonate parent material may exhibit low and high P contents, respectively (Schubert 2002). Additionally, rates of mineral weathering, accumulation of insoluble residues, and soil formation are strongly affected by carbonate parent material purity. Profile *SCH* differed from the other soils by a markedly smaller parent material carbonate content of only 52% (Table S1) compared to at least 95% in the other soils. Furthermore, the parent material P content at *SCH* with 275 µg g^−1^ was about twice as high as at the other carbonate sites (140–150 µg g^−1^) except for *TUT NE* (650 µg g^−1^). At *SCH*, rapid weathering of P- and Fe-rich parent material resulted in formation of 80 cm thick Bw horizons (Table 1) with large stocks of SRO Fe minerals (ferrihydrite) within only 12,000 years. These minerals stored large P_org_ and P_inorg_ stocks (Fig. 2) by strong adsorption, occlusion, and probably also as stable ternary ferrihydrite–PO_4_–Ca complexes (Mendez and Hiemstra 2020). This resulted in small pools of labile P (Fig. 5), low P availability for beech trees (foliar P content 1.13 mg g^−1^; Table 1) and particularly for soil microorganisms (C_mic_/P_mic_ ratio in the Ah horizon: 51; Table 4). We therefore assume that the predominating P-recycling ecosystem nutrition strategy (*ENI*_P_ = –0.4; Table 7) at *SCH* is largely mediated by the O and Ah horizons. Also the dolostone rock at the S-exposed slope at *MAN* (95% carbonate) differed from that at the N-exposed slope (99.6% carbonate) by a markedly larger contribution of non-carbonate compounds (Table S1): Silicon and Al contents were 100 times higher; Fe and K contents were 30 times higher. Yet, both parent materials had almost identical P contents. The increased portion of non-carbonate minerals in the parent rock of the S-exposed profiles resulted (Table 1) in elevated soil contents of total Al, Fe, and K as well as in advanced pedogenesis, as indicated by elevated contents of dithionite- and oxalate-extractable Fe and Al. At *MAN S1,* even a Cambisol with a B horizon has formed within < 12,000 years similar to a Cambisol described by Biermayer and Rehfuess (1985) for a forest site on dolostone rock at 14 km distance. Moreover, and in contrast to the other dolostone sites, P ecosystem nutrition at *MAN S1* had a P-acquiring component in addition to the dominating P-recycling (*ENI*_*P*_: –0.6; other dolostone sites: *ENI*_*P*_: <  < –1; Table 7).

The parent material of profile *TUT NE* differs from that of its SW-exposed counterpart, and also from the pure (> 95%) carbonate parent materials of the other study sites by a more than four times larger P content (650 instead of 150 mg P kg^−1^; Table 1). Consequently, soil P contents (Fig. 1) and stocks (Fig. 2) in profile *TUT NE* were considerably larger than in *TUT SW*. The lower carbonate content in the parent rock of *TUT NE* compared to *TUT SW* was accompanied by three times larger Si, Al, Fe, and K contents (Table S1). This resulted in accelerated pedogenesis, formation of a BA horizon, and lower pH values (Table 1) in *TUT NE* compared to *TUT SW*. These results demonstrate the great importance of the parent material P content for the soil P status on carbonate sites.

### Comparison of sites with carbonate vs. silicate parent material

#### Soil P status (detailed version in Supplementary Information)

(1) Soil P stocks: Total P stocks of the carbonate soils were at the level of the P-poor soils on silicate parent materials (Fig. 2). This can be partly attributed to the low P content of carbonate parent materials, particularly those of high purity, compared to most silicate parent materials (Porder and Ramanchandran 2013). Moreover, chemical weathering of carbonate parent material proceeds much slower than silicate weathering, resulting in low lithogenic P input and low soil accumulation rates of P-retaining sesquioxides and clay minerals. A large part of the P stock in the carbonate forest soils was bound in forest floor SOM. This finding emphasizes the importance of O layer conservation for ecosystem P supply (Ewald 2000; 2005; Prietzel and Ammer 2008; Mellert and Ewald 2014). The relevance of the forest floor to soil P storage and ecosystem P nutrition at carbonate sites decreases with progressing pedogenesis and accumulation of mineral soil material. However, in Central Europe, the Pleistocene glaciations, with few local exceptions, were associated with either complete removal of pre-Pleistocene soils, followed by a reset of pedogenesis in the Holocene, or their conversion into mixed carbonate–silicate soils by (peri)glacial admixing of allochthonous parent materials (*e.g.* loess, till). Thus, mature soils that have formed solely by dissolution of carbonate bedrock and accumulation of non-carbonate residue, such as the *BAE* Cambisol, are extremely rare in Central Europe.

(2) Soil P speciation: In the carbonate-derived soils, a larger portion of total P than in the silicate-derived soils is Ca-bound organic P (Fig. 2). This is probably largely caused by impeded enzymatic cleavage of Ca-P_org_ precipitates (mostly inositol hexaphosphate [IHP] monoesters; Fig. 6; Turner et al. 2002; Wang et al. 2020). Consequently, diester-P/monoester-P ratios were strongly decreased in the carbonate compared to the silicate soils. In summary, our results generally support hypothesis (2) that beech forest soils formed from carbonate rocks differ from those formed from silicate parent material regarding P stocks and P speciation. In general, P stocks of carbonate soils are lower than those of silicate soils, and the dominant P species comprise P_org_-Ca associations and a high share of monoester-P, while in silicate soils diester-P and P_org_-Fe/Al associations are of larger relevance.

(3) Plant and ecosystem P availability: Low beech foliage P contents (Table 1) indicate poor ecosystem P availability at all carbonate sites. Moreover, stocks of plant-available oPO_4_ and C_mic_/P_mic_ ratios in the carbonate soils were at the level of the P-poorest silicate soils *CON* and *LUE* (Fig. 5, Figure S1, Table 4, Table S5). Furthermore, phosphorus enrichment in microbial biomass relative to SOM was much lower in the carbonate than in the silicate soils (Table 4, Table S5). The poor ecosystem P availability of sites with initial carbonate soils is probably caused by strong P incorporation in sparsely soluble Ca–P_org_ precipitates. Ca-bound inositol phosphate is a hardly available P-bearing substrate for microorganisms and plants, resulting in P-rich SOM and large soil P_org_ stocks, whereas at the same time the P supply of soil microorganisms and trees is low.

#### Ecosystem P nutrition strategies of beech forests on carbonate vs. silicate sites

Insufficient P nutrition is a critical factor for growth and vitality of forests on carbonate soils (Ewald, 2000; 2005; Mellert and Ewald 2014). For P-poor silicate sites, Lang et al. (2017) showed that forest ecosystems cope with poor P supply by establishing particular traits of intensive ecosystem-internal P recycling. These traits include plant-internal P-reallocation, but also P recycling within the soil system, *i.e.* intensification of enzymatic P mobilization from SOM, followed by instantaneous re-uptake of mobilized P in the forest floor and the mineral topsoil. Our results in general and in particular the strongly negative *ENI*_*P*_ indices (< –1.3; Table 7) suggest that the Rendzic Leptosols on dolostone at *MAN* were characterized by the same soil traits as at the P-poor silicate sites, *i.e.* pronounced ecosystem P recycling. The accumulation of thick forest floor layers at *MAN*, associated with large values of the P-recycling indicators N4 and N7, was probably caused by the cold and humid site climate (Prietzel et al. 2016c). Ecosystem P acquisition from lithogenic sources as shown for the silicate sites *MIT* and *CON* by Uhlig et al. (2020) was probably restricted at *MAN* by low parent material P contents and weathering rates. Thus, at *MAN* forest floor degradation caused by forest disintegration due to climate warming (Prietzel et al. 2016c) or ungulate pressure (Prietzel and Ammer 2008) results in aggravated ecosystem P shortage and marked changes of soil microorganism communities and nutrient turnover pathways.

**Fig. 10:**
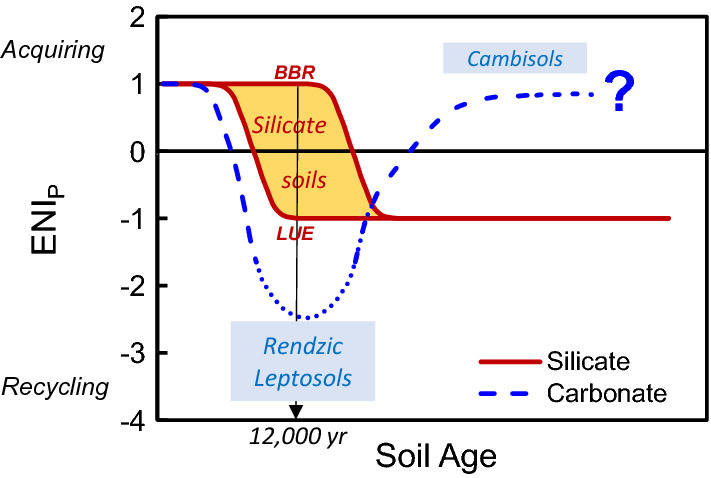
Conceptual model describing ecosystem P nutrition strategy changes of temperate forests on soils formed from carbonate *vs.* silicate bedrock with advancing pedogenesis. *ENI*_*P*_: Ecosystem P Nutrition Index. *BBR: Bad Brückenau*, *LUE: Lüss*

To date, ecosystem P nutrition data for forests on initial carbonate soils are lacking. We assume that, similar to silicate sites (Giguet-Covex et al. 2013; Prietzel et al. 2013), also the continuously recycling ecosystem P stock in Rendzic Leptosols had been acquired from lithogenic sources, *i.e.* by chemical rock weathering, and atmospheric sources, such as mineral dust (Küfmann 2006) and SOM (Zöttl 1965) during initial soil formation and ecosystem succession immediately after deglaciation in the early Holocene. In this context, it is important that hyphae of mycorrhiza and other fungi, but also free soil microorganisms directly access and mine stones and rocks for P (Hinsinger 2001; Stock et al. 2021; Pastore et al. 2022). However, as described in Sect. Soil P status (detailed version in Supplementary Information), soil P input rates by chemical and biological mineral weathering at sites on P-poor carbonate parent material probably are much lower than those at sites on silicate parent material with higher P contents. Thus, it can be assumed that forest ecosystems on initial carbonate soils (similar to those developing on P-poor, quartz-rich silicate parent material) shift from a P-acquiring into a P-recycling nutrition strategy as soon as reasonable amounts of P-containing SOM have been accumulated. In contrast, forests on P-rich silicate parent material may rely for longer time on the P-acquiring nutrition strategy. The systematic change from a predominantly P-acquiring to a predominantly P-recycling nutrition strategy along the geosequence *BBR* (*ENI*_*P*_: 1.0) / *MIT* (0.4) / *VES* (–0.1) / *CON* (–0.6) / *LUE* (–1.0) (Table 7) with decreasing substrate P content (Lang et al., 2017) and soil P stocks (Fig. 8c) may reflect a snapshot taken 12,000 years after onset of soil formation and forest ecosystem succession (Fig. 10). The transformation from initially P-acquiring to ultimately P-recycling nutrition depicted in Fig. 10 is probably caused by accumulation of P-bearing SOM in the forest floor and the mineral topsoil and concomitant gradual replacement of bedrock by P-depleted silicate weathering products (the non-SOM mineral soil fraction) during pedogenesis. Fine earth P contents in the Bw horizons of the profiles *BBR, MIT, VES, CON,* and *LUE* 12,000 years after onset of pedogenesis were 2.0, 0.9, 1.0, 0.4, and 0.2 mg P g^−1^, respectively (Lang et al. 2017), which is only 71%, (exception *MIT* 141%), 43%, 48%, and 50% of the P contents in the respective parent materials (Table S1). The P depletion of the silicate subsoils was probably mainly caused by root P uptake, *i.e.* the initially dominating P-acquiring ecosystem nutrition at all silicate sites.

According to their markedly negative *ENI*_*P*_s, ecosystem P nutrition at all dolostone sites and the limestone site *TUT NE* was dominated by P recycling rather than P acquisition, and a high relevance of soil P_org_ turnover for ecosystem P nutrition, similar to the P-poor silicate sites *CON* and *LUE,* thus supporting hypothesis (2). Yet, we assume that the major pathways of P recycling differ between silicate and carbonate soils at early stages of pedogenesis. At P-poor silicate sites, the prevailing ecosystem P nutrition strategy is characterized by direct biotic recycling of SOM-bound P_org_, which probably is mainly exerted via enzymatic cleavage of SOM-PO_4_ bonds and subsequent uptake of the released oPO_4_ by plant roots, mycorrhiza fungi, and soil microorganisms. In contrast, recycling pathways of SOM-bound P in carbonate soils at early stages of pedogenesis have to include the dissolution of stable Ca-P_org_ (mostly Ca-IHP) precipitates and/or mobilization of calcite-adsorbed IHP (Celi et al. 2000) that had been formed from IHP released during SOM decomposition. Likely because of the continuous re-supply of Ca^2+^ from weathering rock, and unlike at the silicate sites, Ca-P_org_ compounds accumulate and constitute the majority of soil P in carbonate soils with an early stage of pedogenesis (Fig. 2). The forest ecosystems on the Cambisols *MAN S1* and *SCH* according to our results were also characterized by a predominantly recycling P nutrition strategy. However, *ENI*_*P*_s of –0.6 and –0.4, respectively (Table 7) indicate that P-acquiring processes, including microbial (Pastore et al. 2022) and plant uptake of rock and subsoil P at these sites to some extent contribute to ecosystem P nutrition, similar to the silicate site *CON* (*ENI*_*P*_ –0.6; Table 7; Rodionov et al. 2020; Uhlig et al. 2020). It thus can be assumed, in a quantitative sense, that ecosystem P acquisition from lithogenic sources by plants and microorganisms is less effective in soils on P-poor carbonate bedrock (*e.g. MAN*; rock P content 150 mg kg^−1^) compared to most soils on silicate parent materials, which are richer in P (Table S1).

**Fig. 11:**
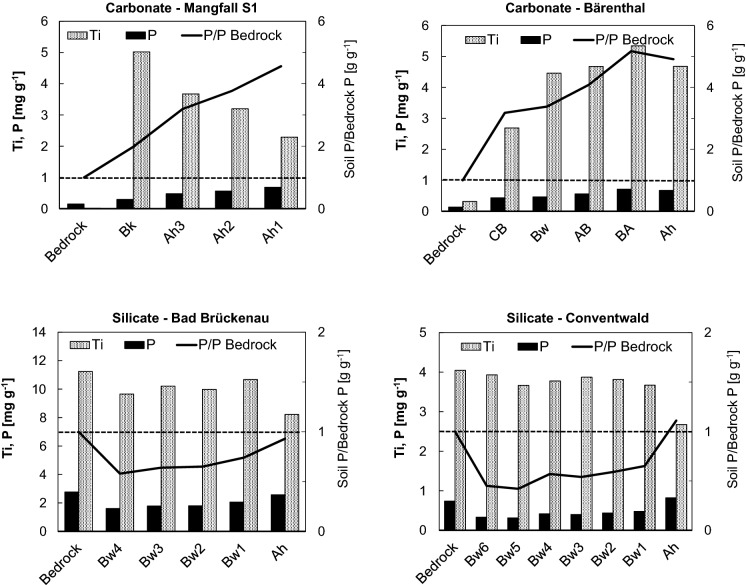
Contents of titanium (Ti, index element for chemical weathering intensity) and phosphorus (P) (left axis) and ratio of soil P/bedrock P (right axis) in different soil horizons of Cambisols formed on carbonate bedrock (*Mangfall Mts. N1*, *Bärenthal*; upper panels) and silicate bedrock (*Bad Brückenau, Conventwald;* lower panels). Dashed line indicates 1:1 ratio of soil P to bedrock P

Intriguingly, site *BAE* with the oldest, pedogenetically most advanced soil in our study (Cambisol with an age > 2.5 Ma) showed the most positive *ENI*_*P*_ (0.9) of all carbonate sites, indicating a predominating P-acquiring ecosystem nutrition strategy. In contrast to silicate sites, forest ecosystem P nutrition on sites with carbonate rock with progressing pedogenesis does obviously not shift systematically from an initial P-acquiring to a P-recycling strategy. Instead, it seems to reverse to a P-acquiring strategy in the “Cambisol phase” after a dominating P-recycling strategy in the previous “Rendzic Leptosol phase”. Of course, the representativeness of our result obtained for *BAE* has to be tested by future investigation of other old Cambisols formed from carbonate rock. Yet, the proposed ecosystem reversal from a predominantly P-recycling to a predominantly P-acquiring nutrition strategy on Cambisols formed from carbonate parent material, which is absent for Cambisols formed from silicate parent material, can be reasonably explained by the different processes responsible for Bw horizon formation in the respective Cambisols. As reported above, at silicate sites, a key pedogenetic process in the formation of Cambisols with Bw horizons is gradual replacement of P-rich silicate rock material by P-poorer silicate weathering products (Fig. 11, lower panels). This P depletion is probably mainly caused by selective apatite dissolution and P mining by soil microorganisms, mycorrhiza fungi, and plant roots, followed by biological P uplift, incorporation of the mobilized P in biomass including partial P removal from the soil, P enrichment and P recycling in the Ah horizon, and to some extent also P losses with the seepage water (Sohrt et al. 2017).

In contrast, in the Cambisols *MAN S1* and *BAE*, the carbonate rock dissolution residue which accumulates in the B horizons in the course of pedogenesis is not depleted, but enriched in P compared to the initial carbonate bedrock (Fig. 11, upper panels). Fine earth P contents in the B horizons of Cambisols *MAN S1* and *BAE* were 0.4 and 0.5 mg P g^−1^ (Table 2), indicating a P enrichment by factor 3 (*BAE*) (Fig. 11) compared to the respective parent materials (P content 0.15 mg g^−1^; Table S1). This P enrichment is mainly caused by the circumstance that in contrast to silicate weathering, during weathering of pure carbonate rock in the course of Cambisol formation the vastly dominating portion of the original rock mass leaves the soil as mobile Ca^2+^, (Mg^2+^), and HCO_3_^−^ with the seepage water. Soil P/rock P content ratios greater than 1 (Fig. 11) prove that lithogenic P (in the carbonate rock mostly present as finely dispersed apatite) which is mobilized in the course of carbonate dissolution, is significantly retained in the carbonate dissolution residue (Al and Fe oxyhydroxides, clay minerals, fine quartz fragments). Phosphorus thus becomes enriched in the weathering residue (rather than depleted as in the silicate soils) compared to the parent material.

TiO_2_ minerals (in soils mostly rutile) are very resistant to chemical weathering, and with progressive chemical weathering of rocks and soils TiO_2_ (and Ti) contents increase due to selective enrichment of these minerals (Milnes and Fitzpatrick 1989; Gupta and Rao 2001). The Ti content in different soil horizons can be used as index of past chemical weathering associated with losses of elements bound in less stable minerals (Sudom and Arnaud 1971; Milnes and Fitzpatrick 1989). Strongly increased Ti contents in the B horizons of the carbonate soils compared to the underlying rock (Fig. 11) indicate considerable historic losses of Ca, Mg, and carbonate during weathering and soil formation. Furthermore, increased P/Ti and P/Fe mass ratios in the Ah horizons of the carbonate soils compared to their respective subsoils (Fig. 11, Figure S4) suggest that plant P uplift leads to additional P topsoil enrichment. Balance calculations (explained in detail in the Supplementary Information) indicate that this phase is associated with ecosystem P leakiness and considerable P ecosystem losses – at least on a time scale of centuries or millennia. One important pathway in this context is P seepage water export. Thus, for a beech forest site with Rendzic Leptosols formed from dolostone in Northern Bavaria, Kaiser et al. (2003) reported an annual export of 40 mg P m^−2^ with the subsoil seepage water. This P export may add up to a total ecosystem loss of 400 g P m^−2^ during 10,000 years of Holocene soil formation, which is more than the total soil P stock in any carbonate soil in our study. Another important pathway of long-term ecosystem P losses is probably topsoil erosion (Alewell et al. 2020). All carbonate soils in our study, including the Cambisols, are characterized by considerable historical *(BAE)* and/or recent (top)soil erosion. According to its soil mineral composition (Stahr and Böcker 2014), *BAE* has largely developed in the Neogene (“Tertiary”), and presumably has lost part of its topsoil material by solifluction in the Pleistocene.

Very likely, the Bw horizon was thicker at the transition Neogene–Pleistocene than today. The entire time of pedogenesis considered, the average rate of soil formation by rock weathering, including complete dissolution of its carbonate fraction, both at *MAN S1* as well as at *BAE* was higher than topsoil material losses by erosion; otherwise, in both profiles no Bw horizons would be present at all. During Bw horizon formation associated with pedogenetic transformation of a Rendzic Leptosol into a Cambisol, P is slowly (because of the low bedrock P content), but steadily released from the weathering carbonate rock into a new-formed deepest Bw horizon section. As explained before, the forest stands at *MAN S1* and *BAE* during the Rendzic Leptosol stadium probably were strongly P-limited. Forest P nutrition depended on the recycling of P that had been acquired by the ecosystem during previous phases of soil formation, and then was stored and recycled in topsoil or forest floor SOM, litter, and plants as well as microbial biomass. At the same time, carbonate dissolution residue with high P content (3 mg g^−1^ P, exceeding even the P content of the basalt at the P-richest silicate site *BBR*; cf. calculation in Supplementary Information) was produced continuously at the boundary layer between the deepest Bw horizon and the carbonate bedrock (“weathering front”).

The P in the carbonate dissolution residue was most likely bound as Ca phosphate (Hinsinger 2001) and/or as ternary Fe oxyhydroxide–PO_4_–Ca complexes (Mendez and Hiemstra 2020). At more advanced stages of pedogenesis, soil pH also in the Bw horizon decreases to values below 6, and soil solution Ca^2+^ concentrations also decrease. Both processes result in P mobilization from secondary Ca-PO_4_ and Ca phytate precipitates (Hinsinger 2001) as well as remobilization of formerly adsorbed inorganic and organic P from dissolving carbonates (Celi et al.^2000^). Thus, in contrast to the Rendzic Leptosol stage of pedogenesis and ecosystem succession, during the Cambisol formation stage of pedogenesis, a large portion of the P that had been released into the soil during previous rock weathering becomes bio-available and probably is rapidly being acquired by plant roots and mycorrhiza fungi. At this stage, forest ecosystems on Cambisols formed from carbonate rock probably gradually (re-)change from a P-recycling into a P-acquiring system (Fig. 10). The additional P injected into the ecosystem P cycle by remobilization of inositol phosphate that had been precipitated as Ca phytate and/or adsorbed to carbonate surfaces in the Rendzic Leptosol stage of soil formation with advancing soil acidification probably markedly increases ecosystem P supply and productivity. Thus, with progressing pedogenesis, forests on carbonate parent material are turning into “pseudo-silicate” systems with (temporarily) high P supply and predominance of P-acquiring ecosystem nutrition. This situation is represented by site *BAE*, whose *ENI*_*P*_ with 0.9 (Table 7) is almost as high as that of the P-richest silicate site *BBR* (1.0). However, plant root P acquisition from the Bw horizon, subsequent plant P uplift, and ultimate deposition of that P on and in the topsoil by litterfall and rhizodeposition result in gradual P depletion, and, thus, P content decrease of the Bw horizon. Simultaneously, with increasing ecosystem P supply P (re)cycling is becoming less tight, and the ecosystem becomes increasingly “leaky” with respect to P. As mentioned before, a major pathway of ecosystem P losses apart from erosion is probably P export with the soil seepage water, particularly as DOP and/or colloid-bound P rather than oPO_4_ (Kaiser et al. 2003; Wang et al. 2020). At the high-elevation site *MAN* with its steep mountain slopes, additionally plant litter and topsoil erosion, probably associated with snow gliding events (Prietzel 2010) contribute to ecosystem P losses. These P losses are continuously replaced by plant P acquisition in the Bw horizon, plant P uplift, and topsoil deposition of litter P, as long as subsoil P is available and rates of soil (Bw) formation and soil P input at the weathering front compensating are same or higher than (top)soil material and P losses. The positive ecosystem P balance at this stage of soil development results in a favorable ecosystem P nutrition status. This is consistent with reports that temperate forests on deep Cambisols formed from carbonate rock generally show good or excellent stand P nutrition (Rehfuess 1990).

In the long run, the positive material balance in developing Cambisols on carbonate rock (*i.e.* the balance of new formation of Bw material at the weathering front in the subsoil minus topsoil losses by erosion) will result in a continuously increasing thickness of the Bw horizon. Then the boundary layer, where P-poor carbonate rock weathers and leaves behind P-rich Bw material will gradually move further down both in absolute terms as well as relative to the soil surface. At some point in time, plant roots may hardly reach it. Ecosystem acquisition of lithogenic P then may become increasingly difficult. At this stage, continuous ecosystem P losses will be associated with progressive P depletion of the rooted soil, with the remaining P bound to soil Fe and Al oxyhydroxides being increasingly less available to plants and soil microorganisms. At this time, ecosystem P supply will deteriorate again, and the system will probably eventually return into P-recycling mode. The ultimate fate of forest ecosystems on soils formed from carbonate bedrock in terms of ecosystem P nutrition thus will be similar to that of forest ecosystems on silicate parent material.

We are aware that the carbonate sites in our study do not represent true chronosequences, and that the presented *ENI*_*p*_ concept is a ranking tool rather than allowing for quantitative assessment of ecosystem P nutrition (see detailed discussion in the Supplementary Information). Nevertheless, the novel information gathered from our study indicates the validity of our hypothesis (3) that the concept of P-acquiring *vs.* P-recycling ecosystems developed for temperate forests at silicate sites by Lang et al. (2016; 2017) is also applicable for carbonate sites. Moreover, it led to the development of a conceptual model describing and comparing the change in forest ecosystem P nutrition strategies (*i.e.* the *ENI*_*p*_) on soils formed from calcareous *vs.* silicate parent material with time and progressing pedogenesis. Our model (Fig. 10) complements the fundamental models describing and explaining soil P and forest ecosystem change on sites with silicate parent material developed by Walker and Syers (1976), Wardle et al. (2004), and Turner et al. (2007; 2013). A novel key feature of our conceptual model is the presence of a second period of P-acquiring ecosystem nutrition in Cambisols formed from carbonate bedrock after an initial phase of dominating P-recycling nutrition when soils are less developed (Rendzic Leptosols). Even if our model may be modified or even refuted in future studies, our study for the first time presents detailed information of soil P and forest ecosystem changes on sites with carbonate parent material, which support large forest areas.

## Conclusions

The P status of temperate forest soils on carbonate parent material at early stages of pedogenesis (Rendzic Leptosols) is characterized by low P stocks and a large fraction of Ca-bound P_org_. At sites with such soils, the P nutrition of beech forests largely depends on tight (re)cycling of P within the forest floor SOM. This highlights the importance of forest floor conservation for ecosystem P nutrition at these sites. Recycling pathways of SOM-bound P in carbonate soils at early stages of pedogenesis and high Ca abundance in the entire profile have to include the dissolution of stable Ca-P_org_ precipitates, which are formed during SOM decomposition and constitute the majority of soil P. With progressing pedogenesis of carbonate soils and formation of Bw horizons, soil P stocks increase. This is due to the formation of Ca-P complexes as well as due to the formation of inorganic P and Al- or Fe-bound P pools, when silicate and Fe oxyhydroxide admixtures in the carbonate parent materials help retain P while more mobile elements become dissolved and lost. Forest P nutrition strategies then return to a second phase of predominately P-acquiring nutrition strategy as it had been at the very onset of soil formation and ecosystem succession. At this stage of ecosystem development, and in contrast to silicate sites, soil acidification and progressing pedogenesis support improved soil P status rather than deteriorating it. Using the novel Ecosystem Phosphorus Nutrition Index (*ENI*_*P*_) allows for assessing the relative contribution of P-acquiring and P-recycling processes for forest ecosystem P nutrition. It proved useful for comprehensive ranking of beech forests on different silicate or carbonate parent materials regarding their ecosystem P nutrition strategy.

## Supplementary Information

Below is the link to the electronic supplementary material.

Supplementary file1 (PDF 840 KB)

## Data Availability

The data will be made available on request.
