# Supplementary material for: Soil phosphorus status and P nutrition strategies of European beech forests on carbonate compared to silicate parent material
Source: Biogeochemistry. 2022 Feb 2;158(1):39–72. doi: 10.1007/s10533-021-00884-7 (PMC8860963; doi:10.1007/s10533-021-00884-7)

**Supplementary Information**

# Soil phosphorus status and P nutrition strategies of European beech forests on carbonate compared to silicate parent material

Jörg Prietzel[1]*, Jaane Krüger[2], Klaus Kaiser[3], Wulf Amelung[4], Sara L. Bauke[4], Michaela A. Dippold[5], Ellen Kandeler[6], Wantana Klysubun[7], Hans Lewandowski[8], Sebastian Löppmann[5,9], Jörg Luster[10], Sven Marhan[6], Heike Puhlmann[11], Marius Schmitt[5], Maja B. Siegenthaler[12], Jan Siemens[13], Sandra Spielvogel[9], Sabine Willbold[8], Jan Wolff[4] and Friederike Lang[2]

**Text content:**

1. Detailed description of methods: Soil sampling, XANES spectroscopy, statistics

2. Detailed version of Section 4.3.1: Comparison of sites with carbonate vs. silicate parent material - Soil P status

3. Calculation of theoretically expected P accumulation in the Bw horizons of Cambisols *MAN S1* and *BAE* by carbonate weathering

4. The Ecosystem Phosphorus Nutrition Index – Application, limitations, perspectives

**Tables S1–S8**

**Figures S1–S4**

## 1. Detailed description of methods: Soil sampling, XANES spectroscopy, statistics

Soil sampling using the Quantitative Pit approach

For quantitative pit establishment, a square wooden frame with an interior lateral length of 50 cm was prepared and registered optical targets for photogrammetric analysis were fixed to the upper side of the frame. The frame was fixed as reference plane to the soil surface using steel pins. The entire organic layer was cut off alongside the inner edge of the frame with a knife. The individual humus layers (Oi, Oe, Oa) were sampled separately by hand. Roots crossing different layers were cut off at the respective layer boundaries and removed to prevent mixing of material from different layers. The mineral soil sampling followed layers representing single diagnostic horizons. If the thickness of a diagnostic horizon exceeded 5 cm for A horizons and 10 cm for B horizons, a new sample layer was started. The procedure was repeated until a maximum sampling depth of 1 m or the consolidated bedrock was reached. We placed all soil material (including rocks and roots) in containers, transported it to the laboratory, and air-dried it at 40°C. Then, we manually separated all soil samples into the following fractions: fine earth (<2 mm), gravel (2–20 mm), stones (>20 mm), coarse roots (>2 mm), fine roots (<2 mm), other soil constituents (*e.g.*, wood, seedlings). After weighing, all fractions were stored dry, cold, and in the dark for further analysis. For soil analysis, we used dried fine earth material. The volume quantification of the different soil layers was carried out based on photogrammetry (Haas et al. 2016). Details concerning the stand representativeness of the QP approach have been discussed earlier (Lang et al. 2017).

**References:**

Haas J, Hagge Ellhöft K, Schack-Kirchner H, Lang F (2016) Using photogrammetry to assess rutting caused by a forwarder—A comparison of different tires and bogie tracks. Soil Tillage Res 163:14–20. https://doi.org/10.1016/j.still.2016.04.008.

Lang F, Krüger J, Amelung W et al (2017) Soil phosphorus supply controls P nutrition strategies of beech forest ecosystems in Central Europe. Biogeochemistry 136:5-29. https://doi.org/10.1007/s10533-017-0375-0.

Soil P speciation by P K-edge XANES spectroscopy

On fine-ground mineral soil subsamples, we acquired P K-edge XANES spectra at beamline 8 of the Synchrotron Light Research Institute (SLRI) in Nakhon Ratchasima, Thailand (Klysubun et al., 2019). Briefly, we spread fine-ground sample powder as thin, homogeneous film on P-free Kapton tape (Lanmar Inc., Northbrook, IL, USA) and mounted the tape on a sample holder. Then we scanned the respective element's X-ray photon energy using an InSb(111) double-crystal monochromator with an energy resolution of $\Delta E/E = 3*10^{-4}$). We recorded all spectra in fluorescence mode with a 13-element germanium detector. To increase fluorescence yield, we placed the sample holder in a 45° angle to the incident monochromatic beam (beam size 12 mm * 1 mm). We constantly purged the sample compartment with He gas to minimize X-ray absorption by air surrounding the sample. The monochromator was calibrated with elemental P ($E_0$ = 2145.5 eV). This was repeated every 12 h, with no indication of $E_0$ movement during the entire beamtime. After calibration, we acquired spectra in the energy range from 2045.5 eV to 2495.5 eV with a 3 s dwell time per energy step. Energy steps were as follows: from 2045.5 to 2105.5 eV and from 2245.5 to 2495.5 eV: energy step of 5 eV; from 2105.5 to 2135.5 eV and from 2195.5 to 2245.5 eV: energy step of 1 eV; from 2135.5 to 2195.5 eV: energy step of 0.25 eV. For each sample, depending on its P concentration, we acquired between two and five spectra. Multiple spectra of a sample always were identical, which rules out artificial sample changes caused by radiation damage. Using the P K-edge XANES spectra, we quantified different P species in all samples applying the protocol of Werner and Prietzel (2015). Using the program *ATHENA* of the software package *DEMETER*, version 0.9.25 (Ravel and Newville 2005), we merged replicate spectra of a given sample after examination for glitches, drifts, noise, and general quality. Spectrum deconvolution was performed using the package *LCF* (version 1.6-6) in the statistical software R. All spectra were initially baseline-corrected from -36 to -15 eV (linear regression) and normalized to an edge-step of 1 from +37 to +58 eV (linear regression) with respect to $E_0$. For linear combination fitting (LCF), we used spectra of the following reference compounds (Prietzel et al. 2016): Crystalline $FePO_4$; amorphous $FePO_4$; crystalline $AlPO_4$; amorphous $AlPO_4$; hydroxy apatite ($Ca_5(OH)(PO_4)_3$); $CaHPO_4$; phytic acid sodium salt hydrate (IHP); Ca phytate; Fe(III) phytate; orthophosphate ($oPO_4$) as well as IHP adsorbed to boehmite, ferrihydrite, and Al-saturated montmorillonite, representing P adsorbed to Al oxyhydroxides, Fe oxyhydroxides, and clay minerals, respectively. To avoid inclusion of too many reference compounds, we refrained from including the reference compounds Mg phosphate and Mg phytate, which in contrast to hydroxy apatite and $CaHPO_4$ are either moderately ($MgHPO_4$) or highly ($MgH_2PO_4$) soluble, or whose solubility decreases only at pH values >7.2 (Mg phytate; [compared to Ca phytate: pH >5.5]; data from Konietzny and Greiner, 2003). To avoid self-absorption and to use standard P concentrations close to those of natural soils, we diluted the standards with fine-ground quartz to 2 mg P $g^{-1}$.

Following the protocol of Werner and Prietzel (2015), we conducted LCF from -14 to +46 eV with respect to $E_0$. The lower energy used for baseline correction varied between -43 and -30 eV (1 eV step), the upper energy between -19 and -9 eV (0.5 eV step) with respect to $E_0$. The lower energy used for normalization varied between +34 and +40 eV (0.5 eV step), the upper energy between +50 and +65 eV (1 eV step) with respect to $E_0$. All spectra were fitted automatically, using baseline-corrected, normalized standard spectra as predictor compounds, allowing for up to six P species being included in the fits. Phosphorus speciation shares <5% of total P were excluded from the result list and LCF was repeated without the respective standard. To improve the accuracy and precision of the LCF results, we used the averages of the five "best" results, i.e. with the smallest R factors as LCF results following the suggestion of Schmieder et al. (2020). Moreover, (i) spectra of P adsorbed to Al or Fe minerals with different degrees of mineral order (*e.g.* ferrihydrite *vs.* goethite), different Al mineral types (*e.g.* gibbsite, Al-saturated montmorillonite, amorphous $Al(OH)_3$), as well as (ii) spectra of inorganic and organic P adsorbed to the same pedogenic mineral are very similar (Prietzel et al. 2016; Gustafsson et al. 2020). Because they therefore are hard to quantify specifically by LCF (Gustafsson et al. 2020), we combined the XANES P speciation data with the results of our wet-chemical determination of organic and inorganic P in the respective samples to yield our final P speciation results. In detail, in topsoil (O, Ah) horizons with pH values <6.5, which were dominated by organic P according to the results yielded with the Saunders and Williams (1955) method, Ca-bound P was assumed to be organic. Inorganic P in these horizons was assumed to be Al-bound, and in some horizons where also Fe-bound P was identified, also Fe-bound. In C and BwC horizons with pH values >7, Ca-bound P was assumed to be organic as well as inorganic, apatite P (Table S1).

**References:**

Gustafsson JP, Braun S, Tuyishime JRM et al (2020) A probabilistic approach to phosphorus speciation of soils using P K-edge XANES spectroscopy with Linear Combination Fitting. Soil Systems 4:26. https://doi.org/10.3390/ soilsystems4020026.

Konietzny U, Greiner R (2003) Phytic acids - Properties and Determination. Encyclopedia Food Sci Nutr 4546–4555. https://doi.org/10.1016/b0-12-227055-x/00922-6

Klysubun W, Tarawarakarn P, Thamsanong N et al (2019) Upgrade of SLRI BL8 beamline for XAFS spectroscopy in a photon energy range of 1–13 keV. Radiat Phys Chem 108145. https://doi.org/10.1016/ j.radphyschem. 2019.02.004.

Prietzel J, Harrington G, Häusler W et al (2016) Reference spectra of important organic and inorganic phosphate binding forms for soil P speciation using synchrotron-based K-edge XANES spectroscopy. J Synchrotron Rad 23:532-544. https://doi.org/10.1107/ S1600577515023 085.

Ravel B, Newville M (2005) ATHENA, ARTEMIS, HEPHAESTUS: data analysis for X-ray absorption spectroscopy using IFEFFIT. J Synchrotron Rad 12:537–541. https://doi.org/10.1107/ S09 09049505012719.

Saunders WMH, Williams EG (1955) Observation on the determination of total organic phosphorus in soils. J Soil Sci 6(2):254–267. https://doi.org/10.1111/j.1365-2389.1955. tb00849.x.

Schmieder F, Gustafsson JP, Klysubun W et al. (2020) Phosphorus speciation in cultivated organic soils revealed by P K-edge XANES spectroscopy. J Plant Nutr Soil Sci 183(3):367-381. https://doi.org/10.1002/ jpln.201900129.

Werner F, Prietzel J (2015) Standard protocol and quality assessment of soil phosphorus speciation by P K-edge XANES spectroscopy. Environ Sci Technol 49:10521-10528. https://doi.org/10.1021/acs.est.5b03096

Statistics

Statistical data analysis (regression, exploratory factor analysis) was performed using the statistical software packages IBM SPSS Statistics (version 24). Linear regression analysis included the calculation of regression equations, as well as $p$ and $R^2$ values between forest floor and total soil P stocks at the different study as independent variables and the respective Ecosystem P Nutrition Index ($ENI_P$) as dependent variable.

**2. Extended version of Section 4.3.1: Comparison of sites with carbonate vs. silicate parent material - Soil P status**

Compared to forest soils on silicate parent material in investigated earlier (*e.g.* Prietzel et al. 2016b; Lang et al. 2017), soils on carbonate parent material differed regarding their P status in three aspects:

**(1) P stocks:** The level of total P stocks of 90–340 g P $m^{-2}$ was at the level of the P-poor soils on silicate parent materials (Figure 2 in main manuscript). At present, no inventory information based on a large number of samples is available that might allow for comparing P stocks of forest soils on carbonate with those on silicate bedrock on global scale. On regional scale, an inventory of 56 forest soils in the 70,000 km² German State of Bavaria (Schubert 2002) showed that six of the ten P-poorest soils had carbonate parent material; the other four P-poor profiles were formed from quartz-rich sand or sandstone. The lower P status of carbonate- than of silicate-derived soils is well in line with the lower P content of most carbonate parent materials, particularly those of high purity, compared to most silicate parent materials (Porder and Ramanchandran 2013). Second, due to intensive proton buffering of carbonates, chemical weathering of carbonate parent material proceeds much slower than silicate weathering. Together with the low P content of most carbonate bedrock types, slow weathering results in low lithogenic P input as well as low soil accumulation rates of P-retaining sesquioxides and clay minerals.

In forest soils on silicate parent material, P stocks were mainly dependent on the general soil nutrient status, including the stocks of total and exchangeable Ca, Mg, and K, which in turn are strongly linked to parent material type (basalt > gneiss > quartz-rich sediments; Lang et al. 2017). In contrast, P stocks of carbonate forest soils (at least of those investigated in our study) were mainly dependent on the forest floor SOM stock. This underpins the well-known important ecological role of O layers in forest ecosystems with shallow carbonate soils (Rendzic Leptosols; *e.g.* Baier et al. 2006; Prietzel and Ammer 2008; Prietzel et al. 2015). Moreover, it emphasizes the importance of O layer conservation for ecosystem vitality in particular with respect to ecosystem P supply (Ewald 2000; 2005; Prietzel and Ammer 2008; Mellert and Ewald 2014). As discussed in Section 4.1 of the main manuscript, the relevance of the forest floor for soil P storage and ecosystem P nutrition at calcareous sites decreases with progressing pedogenesis and accumulation of mineral soil material (i.e. residue of carbonate bedrock dissolution). However, in Central Europe, the Pleistocene glaciations, with few local exceptions, were associated with either complete removal of pre-Pleistocene soils, followed by a reset of pedogenesis in the Holocene, or their conversion into mixed carbonate-silicate soils by (peri)glacial admixing of allochthonous parent

materials (*e.g.* loess, till). Thus, soils formed solely by dissolution of carbonate rocks and accumulation of non-carbonate residue, such as the *BAE* Cambisol, are extremely rare in Central Europe.

**(2) Soil P speciation:** In the carbonate-derived soils, a larger portion of total P (66–90%, on average 77%) was $P_{org}$ than in the soils on silicate bedrock (35–52%, on average 43%) studied by Lang et al. (2017). This was probably partly an effect of impeded enzymatic cleavage of Ca-$P_{org}$ precipitates, as well as of advanced microbial immobilization and cycling of P in microbial residues, which may comprise a majority of SOM constituents (Liang et al. 2019), and can contribute significantly to soil $P_{org}$ forms even in organic surface layers (Wang et al. 2019). With progressing carbonate dissolution and pedogenesis, P-retaining weathering residues consisting of sesquioxides and clay minerals initially present in the carbonate parent material accumulate, and therefore, the amount of $P_{inorg}$ in soils with carbonate parent material as well as its contribution to total soil P increase with pedogenesis (Section 4.1 of main manuscript). Compared to the silicate soils investigated by Lang et al. (2017), the carbonate soils contained larger portions of monoester-P (>85% of $P_{org}$ except for L horizons; Figure 6 in main manuscript; Wang et al. 2020) in the NaOH-EDTA-extractable $P_{org}$ fraction. This monoester-P likely was inositol hexaphosphate (IHP) (Turner et al. 2002). Probably high soil solution Ca concentrations induced effective IHP immobilization as Ca-IHP precipitates in addition to IHP sorption/precipitation on carbonate rock surfaces (McKercher and Anderson 1989, Celi et al. 2000; Celi and Barberis 2007; Prietzel et al. 2016a; Wan et al. 2016). This resulted in marked accumulation of Ca-IHP compounds in carbonate soils with an early stage of pedogenesis, as shown by our P XANES results (Figure 1 in main manuscript), similar to other carbonate soils (Prietzel et al. 2016b). Precipitation of IHP with $Ca^{2+}$ restrains P cycling, and probably is a key factor for the low supply of bioavailable P in carbonate soils at early stages of pedogenesis. Yet, the XANES results also indicated gradual dissolution of Ca-IHP precipitates and accumulation of Al- or Fe-bound $P_{org}$ with progressing pedogenesis, *e.g.* in the topsoil of *BAE*, which has a pH of 5.4. Not only monoester-P, but also diester-P contents were elevated in the carbonate soils: Topsoil contents of both monoester- and diester-P were at the level of the P-richest silicate soil *BBR*, whereas topsoil $P_{inorg}$ contents were markedly smaller. This indicates that diester-P compounds are stabilized and accumulated (Bünemann et al. 2008) in Ca-rich topsoil horizons of carbonate soils. However, diester-P/monoester-P ratios were strongly decreased in the carbonate soils compared to the silicate soils and particularly compared to the P-poor silicate soils *CON* and *LUE* This can be attributed to the fact that on average phosphodiesterase activities were five times higher in the carbonate than in the silicate soils (Table 5 in main manuscript, Table S6), whereas phosphomonoesterase activities were only elevated by <40%, resulting in markedly decreased phosphodiesterase/phosphomonoesterase ratios in the carbonate compared to the silicate soils.

The monoester P accumulation in the carbonate soils thus can be explained by (i) pronounced formation of stable Ca inositol phosphates (*i.e.,* monoesters), (ii) low monoesterase activities, and (iii) the circumstance that enzymatic cleavage of Ca-bound inositol phosphate results in mobilization of the organic inositol moiety than the phosphate group, because binding of inositol phosphate to $Ca^{2+}$ is mediated by the phosphate group. This requires an additional step of Ca-phosphate bond destruction to render the remaining phosphate molecule plant- or ecosystem-available, further hampering the utilization of Ca-bound inositol phosphate by plants and soil microorganisms. In summary, our results generally support hypothesis (1) that temperate forest soils formed from carbonate bedrock differ from those formed from silicate bedrock regarding P stocks and P speciation: They are characterized by smaller total P stocks, predominance of Ca-bound organic P. The circumstance, that temperate forest soils on carbonate bedrock with progressing pedogenesis (*e.g. BAE*) become more similar to silicate soils with respect to their P status, does not really refute hypothesis (1), because old soils, such as BAE, are rare in temperate regions (*e.g.* of Europe and North America), where old soils except for few small spots at most places have been removed by (peri)glacial processes and/or covered with autochthonous material (*e.g*. Loess) during the Pleiszocene, resulting in a re-start of pedogenesis about 12,000 years ago and often of conversion into mixed-parent material soils. Hypothesis (1) thus may be rephrased as follows: Typical temperate forest soils formed from carbonate bedrock in Central Europe are dissimilar to soils formed from silicate bedrock regarding P stocks and P speciation.

**(3) Plant and ecosystem P availability:** Stocks of plant-available $oPO_4$ in the carbonate soils were at the level of the P-poorest silicate soils *CON* and *LUE*, or even smaller in the *MAN N* profiles. Additionally, stocks of labile or moderately labile Hedley P fractions were at the level of the P-poorest silicate soils *CON* and *LUE*, or even smaller in the *MAN N* profiles. Low beech foliage P contents (<1.3 mg $g^{-1}$; except for *MAN S*; Table 1 in main manuscript) also indicate poor ecosystem P availability at all carbonate sites. Moreover, for sites *MAN N1*, *TUT NE*, and *SCH*, where contents of microbial-bound C ($C_{mic}$), N ($N_{mic}$), and P ($P_{mic}$) have been quantified, $C_{mic}/N_{mic}$ ratios (5–7) were considerably smaller compared to those in the silicate-bedrock soils (10–16; Table 4 in main manuscript). This cannot be explained by the circumstance that the microbial communities in the carbonate soils were dominated by bacteria, and those in the acid silicate soils by fungi (Bünemann et al. 2008), which have larger C/N ratios than bacteria (Strickland and Rousk 2010; Mooshammer et al. 2014). According to their PLFA signature, microbial biomass of the carbonate soils in our study was not less, but rather more dominated by fungi than that of the silicate soils, *e.g*. in the Oe horizons (Table S4). Moreover, $C_{mic}/P_{mic}$ ratios in the Ah horizons of the carbonate profiles *MAN N1* and *SCH* (Table 4 in of main manuscript) were at the level of the Ah horizon at the P-poorest silicate site *LUE* (Table S5; Bergkemper et al. 2016). Larger $C_{mic}/P_{mic}$ ratios and markedly smaller $C_{mic}/N_{mic}$ ratios in carbonate

than in the silicate soils suggest the presence of very different microbial communities, with a C:N:P stoichiometry adapted to lower P availability. Microbial stoichiometry was driven by N and P uptake, internal recycling processes during growth, as well as by element storage (Camenzind et al. 2021). In the literature, there is currently an intensive debate whether microbes maintain stable C:N:P ratios independent of resource supply by homeostatic regulation, or whether C:N and C:P ratios of soil microorganisms are more flexible (Bragazza et al. 2021). However, according to Heuck et al. (2015), even the microorganisms in the P-poorest silicate soil at *LUE* are not P-limited. Therefore, P limitation of microbial communities in carbonate soils remains questionable. Yet, the P enrichment in microbial biomass relative to SOM ($C_{org}/P_{org}$ ratios in Ah horizons of carbonate soils: 83–208; Table 4 in main manuscript, silicate soils: 103–905; Table S5) was much lower in carbonate (enrichment factors between 1.6 and 6.5; Table 4 in main manuscript) than silicate soils (enrichment factors of 17 [*BBR*] to 90 [*LUE*]; Table S5). This may be caused by formation of sparsely soluble Ca–$P_{org}$ (inositol phosphate) precipitates in the carbonate soils as described above: Ca-bound $P_{org}$ in carbonate soils is a hardly available P-bearing substrate for microorganisms and plants, resulting in P-rich SOM and large soil $P_{org}$ stocks.

**References;**


Baier R, Ettl R., Hahn C et al (2006) Early development and nutrition of Norway spruce (Picea abies [L.] Karst) seedlings on mineral soil, organic layer, and decayed woody debris origin from dolomite sites of the Bavarian Limestone Alps – a bioassay. Ann For Sci 63:339-348. https://doi.org/10.1051/forest:2006014.

Bergkemper F, Bünemann EK, Hauenstein S et al (2016) An inter-laboratory comparison of gaseous and liquid fumigation based methods for measuring microbial phosphorus ($P_{mic}$) in forest soils with differing P stocks. J Microbiol Meth 128:66-68. https://doi.org/10.1016/j.mimet.2016.07.006.

Bragazza L, Fontana M, Guillaume T et al (2021) Nutrient stoichiometry of a plant-microbe-soil system in response to cover crop species and soil type. Plant Soil 461:517-531. https://doi.org/10.1007/s11104-021-04853-9.

Bünemann EK, Smernik RJ, Marschner P, McNeill AM (2008) Microbial synthesis of organic and condensed forms of phosphorus in acid and calcareous soils. Soil Biol Biochem 40(4):932-946. https://doi.org/10.1016/j.soil bio.2007.11.012.

Camenzind T, Grenz KP, Lehmann J, Rillig MC (2021) Soil fungal mycelia have unexpectedly flexible stoichiometric C:N and C:P ratios. Ecol Lett 24:208–218. https://doi.org/10.1111/ele.13632.

Celi L, Barberis E (2007) Abiotic reactions of inositol phosphates in soil. In: Inositol Phosphates: Linking Agriculture and the Environment (eds BL Turner, AE Richardson, E Mullaney), pp. 207–220. CABI.

Celi L, Lamacchia S, Barberis E (2000) Interaction of inositol phosphate with calcite. Nutrient Cycl Agroecosyst 57:271–277. https://doi.org/10.1023/A:1009805501082.

Ewald J (2000) Is phosphorus deficiency responsible for the low vitality of European beech (Fagus sylvatica L.) in the Bavarian Alps? Forstwiss Centralbl 119:276–296. https://doi.org/10.1007/BF02769143.

Ewald J (2005) Ecological background of crown condition, growth and nutritional status of Picea abies (L.) Karst. In the Bavarian Alps. Eur J Forest Res 124:9–18. https://doi.org/10.1007/s10342-004-0051-5.

Heuck C, Weig A, Spohn M (2015) Soil microbial biomass C:N:P stoichiometry and microbial use of organic phosphorus. Soil Biol Biochem 85:119–129. https://doi.org/10.1016/j.soilbio.2015.02.029.

Lang F, Krüger J, Amelung W et al (2017) Soil phosphorus supply controls P nutrition strategies of beech forest ecosystems in Central Europe. Biogeochemistry 136:5-29. https://doi.org/10.1007/s10533-017-0375-0.

Liang C, Amelung W, Lehmann J, Kästner M (2019) Quantitative assessment of microbial necromass contribution to soil organic matter. Glob Change Biol 11:3578-3590; https://doi.org.10.1111/gcb.14781.

McKercher RB, Anderson G (1989) Organic phosphate sorption by neutral and basic soils. Commun Soil Sci Plant Anal 20(7–8), 723–732. https://doi.org/10.1080/00103628909368112.

Mellert KH, Ewald J (2014) Nutrient limitation and site-related growth potential of Norway spruce (Picea abies [L.] Karst) in the Bavarian Alps. Eur J Forest Res 133:433–451. https://doi.org/10.1007/s10342-013-0775-1.

Mooshammer M, Wanek W, Zechmeister-Boltenstern S et al (2014) Stoichiometric imbalances between terrestrial decomposer communities and their resources: mechanisms and implications of microbial adaptations to their resources. Front Microbiol 5:22. https://doi.org/10.3389/fmicb.2014.00022.

Porder S, Ramachandran S (2013) The phosphorus concentration of common rocks—a potential driver of ecosystem P status. Plant Soil 367(1–2):41–55. https://doi.org/:10.1007/s1110 4012-1490-2

Prietzel J, Ammer C (2008) Montane Bergmischwälder der Bayerischen Kalkalpen: Reduktion der Schalenwild-dichte steigert nicht nur den Verjüngungserfolg, sondern auch die Bodenfruchtbarkeit. Allg Forst Jagdztg 179:104–112.

Prietzel J, Christophel D, Traub C et al (2015) Regional and site related patterns of soil nitrogen, phosphorus, and potassium stocks and Norway spruce nutrition in mountain forests of the Bavarian Alps. Plant Soil 386:151-169. https://doi.org/10.1007/s11104-014-2248-9.

Prietzel J, Harrington G, Häusler W et al (2016a) Reference spectra of important organic and inorganic phosphate binding forms for soil P speciation using synchrotron-based K-edge XANES spectroscopy. J Synchrotron Rad 23:532-544. https://doi.org/10.1107/S1600577515023 085.

Prietzel J, Klysubun W, Werner F (2016b) Speciation of phosphorus in temperate zone forest soils as assessed by combined wet-chemical fractionation and XANES spectroscopy. J Plant Nutr Soil Sci 179:168-185. https://doi. org/0.1002/jpln.201500472.

Schubert A (2002) Bayerische Waldboden-Dauerbeobachtungsflächen–Bodenuntersuchungen. Forstl Forschungs-ber München 187

Strickland MS, Rousk J (2010) Considering fungal:bacterial dominance in soils–Methods, controls, and ecosystem implications. Soil Biol Biochem 42:1385–1395. https://doi.org/10.1016/j.soilbio.2010.05.007.

Wan B, Yan Y, Liu F et al (2016) Surface adsorption and precipitation of inositol hexakisphosphate on calcite: A comparison with orthophosphate. Chem Geol 421:103–111. https://doi.org/10.1016/j.chemgeo.2015.12.004.

Wang L, Amelung W, Prietzel J et al (2019) Transformation of organic phosphorus compounds during 1500 years of organic soil formation in Bavarian Alpine forests – a 31P-NMR study. Geoderma 340:192-205. https://doi.org/10.1016/j.geoderma.2019.01.029.

Wang L, Missong A, Amelung W et al (2020) Dissolved and colloidal phosphorus affect P cycling in calcareous forest soils. Geoderma 375:114507. https://doi.org/10.1016/j.geoderma.2020.114507.

**3. Calculation of theoretically expected P accumulation in the Bw horizons of the Cambisols *Mangfallgebirge (MAN) S1* and *Bärenthal (BAE)* by carbonate weathering**

*Theoretically expected vs. measured P contents in Bw horizons of MAN S1 and BAE*

At sites *MAN S1* and *BAE* the bedrock has a Ca/Mg carbonate (dolomite) content of 950 mg $g^{-1}$ and a P content of 0.15 mg $g^{-1}$ (Table S1). Thus, rock weathering, assuming complete dissolution of its dolomite fraction and subsequent removal of the carbonate dissolution products $Ca^{2+}$, ($Mg^{2+}$; *MAN*) and $HCO_3^-$ with the soil seepage water, and assuming that the non-carbonate weathering products remain in the soil as "carbonate dissolution residue", results in a remaining carbonate solution residue mass of 5 g from originally 100 g bedrock, or of 0.05 g per 1 g of original dolomite bedrock, corresponding to a reduction in soil volume by a factor of 20. The expected P content of the carbonate dissolution residues in soils *MAN S1* and *BAE*, assuming absence of P loss during carbonate weathering, is thus concentrated by a maximum of up to a factor of 20, thus amounting to 20 * 0.15 mg P $g^{-1}$ = 3 mg P $g^{-1}$. This is 7.5 times as much as the measured P content of the Bw horizon (0.4 mg $g^{-1}$) in profile *MAN S1*, and 6 times as much as the measured P content of the Bw horizon (0.5 mg $g^{-1}$) in profile *BAE, i.e.* there was considerable P uptake and loss from these horizons.

*Theoretically expected vs. measured fine earth P stocks in Bw horizons of MAN S1 and BAE*

At sites *MAN S1* and *BAE* the parent material has a Ca carbonate (calcite) content of 950 mg $g^{-1}$ and a P content of 0.15 mg $g^{-1}$ (Table S1). The densities of dolomite (bedrock of *MAN S1*) and calcite (parent material of *BAE*) are 2.9 g $cm^{-3}$ and 2.7 g $cm^{-3}$, respectively. The density of accessory silicates (5% of total rock mass at both sites) is 2.75 g $cm^{-3}$ and thus similar to carbonate densities. Total thicknesses of the Bk/Bw horizons of profiles *MAN S1* and *BAE* are 15 cm and 20 (BwAh) +16 (Bw) = 36 cm, respectively (Table 1). Fine earth bulk densities, corrected for stone content, in the Bk/Bw horizons of *MAN S1* and *BAE* are 1.87 and 1.1 g $cm^{-3}$ (Stahr and Böcker 2014), respectively.

At site *MAN S1*, dissolution of 1 m carbonate rock, using the data reported above would result in a Bk horizon consisting of carbonate dissolution residue with a thickness of (100–95) * 2.9/1.87 = 5 * 2.9/1.87 cm = 7.75 cm. Thus, to form a Bk horizon with a thickness of 15 cm (which is present at *MAN S1*), a column of 15/7.75 * 1 m = 1.93 m dolomite rock must have been weathered. This is a conservative estimate, assuming no loss of carbonate dissolution residue in colloidal or dissolved form with the seepage water. At a dolomite density of 2.9 g $cm^{-3}$, this corresponds (slope effects on horizontal surface area projection being neglected) to a carbonate rock mass of 2.9 [g $cm^{-3}$] * 193 [cm] [1 $cm^{-2}$] per $cm^2$ soil surface = 560 g $cm^{-2}$ = 5,600 kg $m^{-2}$. The amount of P contained in that

rock mass (at a P content of 0.15 mg $g^{-1}$) is 0.15 * $10^{-3}$ * 5,600 kg $m^{-2}$ = 0.84 kg P $m^{-2}$ or 8,400 kg P $ha^{-1}$. However, the total soil P stock of *MAN S1* is only 4,000 kg $ha^{-1}$, and the P stock in the subsoil (Bk) is even only about 1000 kg $ha^{-1}$ (Figure 2). Thus, if one reasonably assumes that the P content of the rock whose residues have produced the existing Bw horizon is similar to the P content of the rock analyzed in our study, the majority of the P amount released from the parent rock during carbonate dissolution must have been lost during pedogenesis leading to the formation of the Bk horizon at *MAN S1*. Probable reasons for that loss are discussed below.

At site *BAE*, dissolution of 1 m carbonate rock, using the data reported above would result in a Bw horizon consisting of carbonate dissolution residue with a thickness of (100–95) * 2.7/1.1 cm = 5 * 2.7/1.1 cm = 12.3 cm. Thus, to form a Bw horizon with a thickness of 36 cm (which is present at *BAE*), a column of 36/12.3 * 1 m = 2.93 m dolomite rock must have been weathered. Again, this is a conservative estimate, assuming no loss of carbonate dissolution residue with the seepage water. At a calcite density of 2.7 g $cm^{-3}$, this corresponds to a carbonate rock mass of 2.7 [g $cm^{-3}$] * 293 [cm] [1 $cm^{-2}$] per $cm^2$ soil surface = 792 g $cm^{-2}$ = 7,920 kg $m^{-2}$. The amount of P contained in that rock mass (at a P content of 0.15 mg $g^{-1}$) is 0.15 * $10^{-3}$ * 7,920 kg $m^{-2}$ = 11.9 kg P $m^{-2}$ or 11,900 kg P $ha^{-1}$. However, the total soil P stock of *BAE* is only 3,200 kg $ha^{-1}$, and the P stock in the subsoil (Bw) is even only about 2,400 kg $ha^{-1}$. (Figure 2). Thus, assuming that the P content of the parent rock whose residues have produced the existing Bw horizons is similar to the P content of the rock analyzed in our study, >70% of the P amount released from the parent rock during carbonate dissolution must have been lost during the pedogenesis that lead to the formation of the Bv horizons at *BAE*.

**4. The Ecosystem Phosphorus Nutrition Index – Application, limitations, perspectives**

The $ENI_P$ approach seems to be useful for a comprehensive evaluation and ranking of temperate beech forest sites on silicate and carbonate rock regarding their ecosystem P nutrition strategy, and even allowed the development of a new conceptual model (Figure 10). Yet, it must be emphasized that the $ENI_P$ values calculated for our study sites are interval-scaled rather than ratio-scaled – the scale was defined arbitrarily (even though based on good reasons) by the end-members *BBR* and *LUE* which had been identified as "most P acquiring" and "most P recycling" silicate sites, respectively, by Lang et al. (2017). Application of that scale to the carbonate sites in our study generally yielded reasonable results. Yet, $ENI_P$s down to –7.1 for site *MAN S2* considerably extended the original scale (ranging from –1 to +1) in the negative (*i.e.* P-recycling) direction. As mentioned before, data on the P-recycling indicator N6 were only available for two of the eight carbonate sites, and data on the P-acquiring indicators N2 and N3 were only available for four carbonate sites (Table 7). These data deficits theoretically may have confounded the $ENI_P$s calculated for the respective sites. We therefore repeated our $ENI_P$ calculations for all sites, omitting N6 (Table S7), or N2, N3, and N6 (Table S8). The $ENI_P$s calculated without inclusion of N6 were very similar to those calculated with inclusion of N6: Without inclusion, profiles *MAN N1* and *N2* both had $ENI_P$s of –2.0, and an $ENI_P$ for *TUT NE* of –0.7 instead of –0.5 indicated a somewhat larger influence of P-recycling ecosystem nutrition (Table S7). Also, the $ENI_P$s calculated without inclusion of N2, N3, and N6 (Table S8) did not markedly change the respective ecosystem P nutrition strategies identified for the different sites, nor their sequence with respect to recycling *vs.* acquiring P nutrition. Alternatively, we completed the indicator matrix for all dolostone and limestone sites by replacing missing values with the arithmetic mean of the respective indicator, distinguishing between dolostone and limestone sites. The $ENI_P$s calculated this way for the dolostone sites were (original $ENI_P$ without indicator replacement in brackets) –5.3 (–7.1) for *MAN S2,* –1.8 (–2.0) for *MAN S1,* –1.6 (–1.4) for *MAN N1,* and –0.4 (–0.6) for *MAN S2*. Thus, $ENI_P$s for the dolostone forests seem not markedly biased by missing values for single variables, and it can be reasonably assumed that the *MAN* forests on shallow Rendzic Leptosols with P-poor dolostone rock and thick forest floor layers were more dominated by P-recycling ecosystem P nutrition traits than the P-poor Dystric Cambisol *LUE*. Thus, $ENI_P$ values <1 are reasonable for the *MAN* Rendzic Leptosol sites. For the limestone sites, $ENI_P$s after replacement of missing values were –0.5 (–0.5) for *TUT NE,* –0.4 (0.2) for *TUT SW,* –0.3 (–0.4) for *SCH,* and –0.2 (0.9) for *BAE*. Thus, at *BAE,* where only N1, N4, N7 were available for $ENI_P$ calculation, the lack of data for the other indicators may have resulted in erroneous assignment of that site to a dominating P acquiring nutrition strategy (Table 7) instead of more likely coexistence of P-acquiring and P-recycling strategies. Nevertheless, site *BAE* with its oldest soil according to our different calculations always was the least P-recycling and most P-acquiring forest of all carbonate sites. In this context, it must be pointed out

that the $ENI_P$ concept in its present state provides a ranking tool rather than a quantitative assessment of ecosystem P nutrition. Thus, it surely cannot be claimed that *MAN S2* is a seven times more P-recycling ecosystem than *LUE*. The $ENI_P$ concept itself as well as the variables it is currently based on surely represent the first stage of a longer development process. Concept and variables used have to be checked rigorously for additional soils on silicate and carbonate parent material in future studies, and improved, if necessary. Moreover, the applicability of the concept for other tree species and climate regions has to be tested. Yet, we suggest that our concept to integrate a large set of variables describing ecosystem P nutrition (acquisition, recycling) strategies into one well-defined $ENI_P$ for comparing forest ecosystems with respect to their dominating P nutrition strategy is a promising tool for forest ecology and biogeochemistry.

**Table S1:** Content of Ca(Mg) carbonate and of important elements in rock samples from the calcareous study sites and the silicate sites investigated by Lang et al. *(*2017).

| Site | Carbonate | Si | Ti | Al | Fe | Mn | Ca | Mg | K | Na | P |
|---|---|---|---|---|---|---|---|---|---|---|---|
| | ---------------------- | ---------------------- | ---------------------- | ---------------------- | ---------------------- | ---------------------- g $kg^{-1}$ ---------------------- | ---------------------- | ---------------------- | ---------------------- | ---------------------- | ---------------------- |
| *Calcareous bedrock sites* | | | | | | | | | | | |
| Mangfallgebirge S1 | 950 | 13.0 | 0.27 | 5.61 | 2.59 | 0.05 | 210 | 122 | 2.57 | 1.41 | 0.148 |
| Mangfallgebirge N2 | 996 | 0.14 | 0.02 | 0.05 | 0.14 | 0.03 | 222 | 131 | 0.08 | 1.34 | 0.140 |
| Tuttlingen SW | 985 | 3.79 | 0.10 | 1.11 | 0.84 | 0.05 | 388 | 3.08 | 0.42 | 1.48 | 0.144 |
| Tuttlingen NE | 960 | 10.8 | 0.19 | 3.33 | 2.52 | 0.16 | 381 | 3.20 | 1.58 | 1.48 | 0.650 |
| Schänis | 518 | 204 | 0.59 | 9.95 | 11.6 | 0.43 | 198 | 3.02 | 2.82 | 1.71 | 0.271 |
| Bärenthal | 949 | ND | ND | 6.29 | 12.9 | 0.14 | 400 | 1.89 | 0.00 | ND | 0.150 |
| *Silicate bedrock sites* | | | | | | | | | | | |
| Bad Brückenau | 0 | 197 | 12.3 | 55.5 | 82.9 | 1.57 | 68.3 | 107.3 | 8.27 | 15.0 | 2.78 |
| Mitterfels | 0 | 319 | 4.07 | 75.2 | 40.7 | 0.90 | 11.2 | 13.4 | 16.5 | 20.5 | 0.642 |
| Vessertal | 0 | 274 | 6.15 | 87.4 | 37.4 | 0.97 | 8.14 | 21.2 | 55.6 | 23.9 | 2.29 |
| Conventwald | 0 | 296 | 5.03 | 88.2 | 45.7 | 0.53 | 2.95 | 16.7 | 26.6 | 17.5 | 0.825 |
| Lüss | 0 | 385 | 2.91 | 29.6 | 25.6 | 0.39 | 16.0 | 10.6 | 10.9 | 6.53 | 0.401 |

**Table S2:** Content of different P species in the investigated soils. $P_{org}$ and $P_{inorg}$ determined according to Saunders and Williams (1955). Percentages of Ca-bound P, Al-bound P, and Fe-bound P determined by synchrotron-based XANES spectroscopy. Error of XANES speciation results <10% (Werner and Prietzel, 2017).

| *Site* | Horizon | $P_{org}$ | $P_{inorg}$ | $P_{org}$ / ($P_{org}$+$P_{inorg}$) | $NaHCO_3$-extractable-P | | --------- Organic P ---------- | | | ---------- Inorganic P --------- | | | R factor |
|---|---|---|---|---|---|---|---|---|---|---|---|---|---|
| | | | | | $oPO_4$-P | Total P | Ca-bound | Al-bound | Fe-bound | Ca-bound | Al-bound | Fe-bound | XANES |
| | | ------ mg $g^{-1}$ ------ | | | --------- mg $kg^{-1}$ -------- | | ------------------------------ Percentage of total P ---------------------------- | | | | | | LCF |
| *Mangfall-gebirge S1* | Oi | 0.383 | 0.085 | 0.82 | 63.2 | 51.4 | ND | ND | ND | ND | ND | ND | ND |
| | Oe | 0.671 | 0.201 | 0.77 | 91.4 | 79.7 | ND | ND | ND | ND | ND | ND | ND |
| | Oa | 0.654 | 0.195 | 0.77 | 45.2 | 45.5 | ND | ND | ND | ND | ND | ND | ND |
| | Ah1 | 0.718 | 0.135 | 0.84 | 18.7 | 26.6 | 84 | | | | 16 | | 0.0059 |
| | Ah2 | 0.706 | 0.108 | 0.87 | 14.3 | 26.0 | 87 | | | | 13 | | 0.0071 |
| | Ah3 | 0.598 | 0.125 | 0.83 | 16.0 | 23.8 | 83 | | | | 17 | | 0.0046 |
| | Ah4 | 0.406 | 0.087 | 0.82 | 9.3 | 21.2 | 82 | | | | 18 | | 0.0052 |
| | Bk | 0.217 | 0.048 | 0.82 | 2.9 | 9.3 | 54 | | | | | 18 | 0.0250 |
| *Mangfall-gebirge S2* | Oi | 0.274 | 0.103 | 0.73 | 96.7 | 75.0 | ND | ND | ND | ND | ND | ND | ND |
| | Oe | 0.576 | 0.252 | 0.70 | 144.2 | 132.3 | ND | ND | ND | ND | ND | ND | ND |
| | Oa | 0.734 | 0.137 | 0.84 | 51.5 | 58.7 | ND | ND | ND | ND | ND | ND | ND |
| | Ah | 0.717 | 0.139 | 0.84 | 15.3 | 21.8 | 64 | 20 | | | 16 | | 0.0015 |
| | CA | 0.703 | 0.105 | 0.87 | 11.3 | 16.2 | 54 | 33 | | | 13 | | 0.0006 |
| *Mangfall-gebirge N1* | Oi | 0.404 | 0.108 | 0.79 | 98.4 | 11.4 | ND | ND | ND | ND | ND | ND | ND |
| | Oe | 0.651 | 0.145 | 0.82 | 88.0 | 88.0 | ND | ND | ND | ND | ND | ND | ND |
| | Oa | 0.952 | 0.163 | 0.85 | 60.2 | 59.0 | ND | ND | ND | ND | ND | ND | ND |
| | Ah | 0.955 | 0.119 | 0.89 | 28.5 | 33.3 | 89 | | | | 11 | | 0.0044 |
| | CA | 0.868 | 0.070 | 0.93 | 7.0 | 10.8 | 80 | | 13 | | | 7 | 0.0023 |
| *Mangfall-gebirge N2* | Oi | 0.416 | 0.091 | 0.82 | 104.2 | 84.2 | ND | ND | ND | ND | ND | ND | ND |
| | Oe | 0.652 | 0.115 | 0.85 | 82.8 | 74.3 | ND | ND | ND | ND | ND | ND | ND |
| | Oa | 1.088 | 0.144 | 0.88 | 54.8 | 57.5 | ND | ND | ND | ND | ND | ND | ND |
| | Ah | 1.097 | 0.136 | 0.89 | 21.7 | 24.3 | 63 | 26 | | | 11 | | 0.0011 |
| | CA | 0.935 | 0.101 | 0.90 | 5.9 | 10.0 | 62 | 28 | | | 10 | | 0.0012 |

**Table S2** (continued).

| *Site* | Horizon | $P_{org}$ | $P_{inorg}$ | $\frac{P_{org}}{(P_{org}+P_{inorg})}$ | $NaHCO_3$-extractable-P | | --------- Organic P ---------- | | | ---------- Inorganic P --------- | | | R factor |
|---|---|---|---|---|---|---|---|---|---|---|---|---|---|
| | | | | | $oPO_4$-P | Total P | Ca-bound | Al-bound | Fe-bound | Ca-bound | Al-bound | Fe-bound | XANES |
| | | ------ mg $g^{-1}$ ------ | | | --------- mg $kg^{-1}$ -------- | | ----------------------------- Percentage of total P ----------------------------- | | | | | | LCF |
| *Tuttlingen* | Oi | 0.676 | 0.181 | 0.79 | 117.9 | 140.8 | ND | ND | ND | ND | ND | ND | ND |
| *NE* | Oe | 0.826 | 0.318 | 0.72 | 130.5 | 149.4 | ND | ND | ND | ND | ND | ND | ND |
| | Ah1 | 0.837 | 0.175 | 0.83 | 29.6 | 46.0 | 27 | | 56 | | | 17 | 0.0023 |
| | Ah2 | 0.812 | 0.164 | 0.83 | 16.1 | 31.0 | 18 | 26 | 39 | | 20 | | 0.0005 |
| | Ah3 | 0.761 | 0.124 | 0.86 | 16.2 | 30.5 | 42 | | 44 | | | 14 | 0.0067 |
| | Ah4 | 0.671 | 0.100 | 0.87 | 17.1 | 44.9 | 49 | 38 | | | 13 | | 0.0005 |
| | BA | 0.621 | 0.104 | 0.86 | 13.5 | 36.1 | 14 | | 72 | 14 | | | 0.0029 |
| | C1 | 0.621 | 0.142 | 0.81 | 6.7 | 16.8 | 18 | | 63 | 19 | | | 0.0043 |
| | C2 | 0.629 | 0.199 | 0.76 | 3.2 | 12.0 | 34 | | 42 | 19 | 5 | | 0.0055 |
| *Tuttlingen* | Oi | 0.278 | 0.255 | 0.52 | 186.6 | 237.3 | ND | ND | ND | ND | ND | ND | ND |
| *SW* | Oe | 0.611 | 0.246 | 0.71 | 171.3 | 207.0 | ND | ND | ND | ND | ND | ND | ND |
| | Ah1 | 0.717 | 0.192 | 0.79 | 30.0 | 72.1 | 67 | 12 | | | 21 | | 0.0009 |
| | Ah2 | 0.781 | 0.130 | 0.86 | 24.0 | 63.0 | 67 | 19 | | | 14 | | 0.0009 |
| | Ah3 | 0.796 | 0.136 | 0.85 | 16.7 | 38.0 | 63 | 15 | 7 | | 15 | | 0.0009 |
| | CA1 | 0.643 | 0.121 | 0.84 | 7.5 | 22.7 | 64 | 20 | | | 16 | | 0.0007 |
| | CA2 | 0.610 | 0.127 | 0.83 | 8.7 | 25.5 | 66 | 17 | | | 17 | | 0.0005 |
| | CA3 | 0.574 | 0.156 | 0.79 | 4.0 | 13.1 | 65 | 5 | 9 | | 21 | | 0.0009 |
| | CA4 | 0.477 | 0.188 | 0.72 | 3.0 | 9.5 | 68 | 4 | | | 28 | | 0.0005 |
| *Bärenthal* | OiOe | 0.731 | 0.085 | 0.90 | ND | ND | ND | ND | ND | ND | ND | ND | ND |
| | Ah1 | 0.591 | 0.054 | 0.92 | ND | ND | 15 | 52 | 25 | | 5 | 3 | 0.0006 |
| | AB | 0.501 | 0.052 | 0.91 | ND | ND | | 39 | 52 | | 4 | 5 | 0.0015 |
| | BA | 0.404 | 0.048 | 0.89 | ND | ND | | 65 | 24 | | 11 | | 0.0007 |
| | Bw | 0.247 | 0.092 | 0.73 | ND | ND | 33 | 19 | 21 | | | 27 | 0.0084 |
| | CB | 0.269 | 0.099 | 0.73 | ND | ND | 37 | 34 | 2 | | 21 | 6 | 0.0045 |
| *Schänis* | Oi | 0.460 | 0.174 | 0.73 | 108.6 | 114.0 | ND | ND | ND | ND | ND | ND | ND |
| | Oe | 0.879 | 0.350 | 0.72 | 161.2 | 123.4 | ND | ND | ND | ND | ND | ND | ND |
| | Ah1 | 0.781 | 0.141 | 0.85 | 15.9 | 170.4 | 73 | 6 | 6 | | 10 | 3 | 0.00xx |
| | Ah2 | 0.674 | 0.103 | 0.87 | 6.9 | 29.9 | 78 | 5 | 5 | | 11 | 2 | 0.00xx |
| | Ah3 | 0.615 | 0.093 | 0.87 | 4.7 | 18.2 | 54 | | 33 | | | 13 | 0.0023 |
| | Bw1 | 0.513 | 0.078 | 0.87 | 3.0 | 0.1 | 60 | | 27 | | | 13 | 0.0046 |
| | Bw2 | 0.322 | 0.072 | 0.82 | 1.9 | 0.1 | 25 | | 57 | 17 | | | 0.0057 |
| | 2Bw | 0.300 | 0.277 | 0.52 | 3.8 | 7.7 | 31 | | 21 | 45 | | 3 | 0.0168 |
| | 3CB | 0.359 | 0.265 | 0.58 | 3.3 | 9.8 | 39 | | 19 | 42 | | | 0.0027 |

**Table S3:** Contents of different Hedley P fractions (mg g$^{-1}$) in the investigated soils.

| | | Pinorg Resin | Pinorg $NaHCO_3$ | Porg $NaHCO_3$ | Pinorg 1M HCl | Pinorg NaOH | Porg NaOH | P HCl conc | P residual |
|---|---|---|---|---|---|---|---|---|---|
| | | *Labile* $P_{inorg}$ | *Labile* $P_{inorg}$ | *Labile* $P_{org}$ | *Moderately labile* $P_{inorg}$ | *Moderately labile* $P_{inorg}$ | *Moderately labile* $P_{org}$ | *Stable P* | *Stable P* |
| *Mangfall-gebirge S1* | Ah1 | 0.046 | 0.083 | 0.171 | 0.014 | 0.018 | 0.039 | 0.194 | 0.277 |
| | Ah2 | 0.044 | 0.075 | 0.163 | 0.013 | 0.017 | 0.036 | 0.184 | 0.246 |
| | Ah3 | 0.042 | 0.039 | 0.142 | 0.009 | 0.017 | 0.028 | 0.162 | 0.215 |
| | Ah4 | 0.032 | 0.026 | 0.120 | 0.008 | 0.012 | 0.025 | 0.144 | 0.219 |
| | Bk | 0.019 | 0.007 | 0.065 | 0.001 | 0.005 | 0.009 | 0.103 | 0.166 |
| *Mangfall-gebirge N1* | Ah | 0.036 | 0.141 | 0.215 | 0.042 | 0.017 | 0.038 | 0.379 | 0.455 |
| | CA | 0.008 | 0.021 | 0.067 | 0.022 | 0.007 | 0.007 | 0.202 | 0.486 |
| *Tuttlingen NE* | Ah1 | 0.041 | 0.080 | 0.247 | 0.026 | 0.020 | 0.040 | 0.292 | 0.204 |
| | Ah2 | 0.038 | 0.043 | 0.196 | 0.025 | 0.018 | 0.038 | 0.283 | 0.388 |
| | Ah3 | 0.030 | 0.037 | 0.168 | 0.022 | 0.012 | 0.026 | 0.264 | 0.397 |
| | Ah4 | 0.020 | 0.019 | 0.107 | 0.017 | 0.007 | 0.013 | 0.264 | 0.315 |
| | BA | 0.016 | 0.013 | 0.096 | 0.026 | 0.007 | 0.014 | 0.261 | 0.315 |
| | C1 | 0.012 | 0.011 | 0.050 | 0.062 | 0.005 | 0.008 | 0.252 | 0.426 |
| | C2 | 0.010 | 0.008 | 0.054 | 0.105 | 0.004 | 0.007 | 0.272 | 0.335 |
| *Bärenthal* | Ah1 | 0.039 | 0.059 | 0.203 | 0.011 | 0.005 | 0.039 | 0.201 | 0.269 |
| | AB | 0.036 | 0.028 | 0.179 | 0.009 | 0.001 | 0.039 | 0.195 | 0.267 |
| | BA | 0.019 | 0.007 | 0.068 | 0.007 | 0.006 | 0.008 | 0.230 | 0.372 |
| | Bw | 0.017 | 0.005 | 0.028 | 0.017 | 0.001 | 0.005 | 0.216 | 0.282 |
| | CB | 0.008 | 0.005 | 0.026 | 0.036 | 0.000 | 0.006 | 0.196 | 0.305 |
| *Schänis* | Ah1 | 0.034 | 0.046 | 0.154 | 0.043 | 0.014 | 0.031 | 0.287 | 0.360 |
| | Ah2 | 0.020 | 0.016 | 0.120 | 0.026 | 0.008 | 0.015 | 0.286 | 0.310 |
| | Ah3 | 0.017 | 0.012 | 0.098 | 0.022 | 0.004 | 0.012 | 0.279 | 0.350 |
| | Bw1 | 0.012 | 0.008 | 0.072 | 0.018 | 0.004 | 0.006 | 0.237 | 0.296 |
| | Bw2 | 0.011 | 0.006 | 0.055 | 0.013 | 0.004 | 0.004 | 0.184 | 0.238 |
| | 2Bw | 0.007 | 0.005 | 0.029 | 0.166 | 0.003 | 0.005 | 0.173 | 0.181 |
| | 3CB | 0.010 | 0.008 | 0.032 | 0.158 | 0.003 | 0.006 | 0.192 | 0.205 |

**Table S4:** Contents of total, fungal, and bacterial Phospholipid Fatty Acids (PLFA) in the study soils with calcareous and silicate parent material.

| | Total PLFA | Fungal PLFA | PLFA Gram-positive bacteria | PLFA Gram-negative bacteria | Bacterial PLFA | Fungal PLFA/ (Fungal+bacterial PLFA) |
|---|---|---|---|---|---|---|
| | ------------------------------ nmol $g^{-1}$ soil | | | | --------------------------------- | (mol/mol) |
| **Oe horizons** | | | | | | |
| *Calcareous soils* | | | | | | |
| MAN N | 1498 | 139 | 192 | 143 | 335 | 0.29 |
| MAN S | 1455 | 159 | 199 | 89 | 288 | 0.36 |
| TUT SW | 1360 | 323 | 151 | 43 | 195 | 0.62 |
| TUT NE | 850 | 150 | 113 | 32 | 145 | 0.51 |
| SCH | 1379 | 320 | 148 | 29 | 177 | 0.64 |
| BAE | 1177 | 249 | 124 | 67 | 190 | 0.57 |
| *Mean value Calcareous soils* | | | | | | **0.50** |
| *Silicate soils* | | | | | | |
| MIT | 991 | 86 | 133 | 34 | 167 | 0.34 |
| VES | 1200 | 130 | 195 | 58 | 253 | 0.34 |
| LUE | 1106 | 121 | 151 | 37 | 188 | 0.39 |
| *Mean value Silicate soils* | | | | | | **0.36** |
| **Oa horizons** | | | | | | |
| *Calcareous soils* | | | | | | |
| MAN N | 741 | 31 | 140 | 60 | 200 | 0.13 |
| MAN S | 1064 | 48 | 215 | 50 | 265 | 0.15 |
| *Mean value Calcareous soils* | | | | | | **0.14** |
| *Silicate soils* | | | | | | |
| MIT | 1058 | 66 | 234 | 50 | 285 | 0.19 |
| VES | 410 | 14 | 89 | 27 | 116 | 0.11 |
| CON | 672 | 18 | 131 | 38 | 168 | 0.10 |
| LUE | 478 | 32 | 105 | 25 | 131 | 0.19 |
| *Mean value Silicate soils* | | | | | | **0.15** |
| **Ah horizons** | | | | | | |
| *Calcareous soils* | | | | | | |
| MAN N | 327 | 8 | 79 | 23 | 102 | 0.07 |
| MAN S | 551 | 16 | 138 | 27 | 165 | 0.09 |
| TUT SW | 337 | 40 | 53 | 19 | 72 | 0.36 |
| TUT NE | 347 | 27 | 64 | 16 | 80 | 0.25 |
| SCH | 207 | 6 | 42 | 9 | 51 | 0.11 |
| BAE | 302 | 14 | 65 | 17 | 83 | 0.14 |
| *Mean value Calcareous soils* | | | | | | **0.17** |
| *Silicate soils* | | | | | | |
| BBR | 512 | 23 | 115 | 40 | 155 | 0.13 |
| MIT | 243 | 6 | 57 | 12 | 69 | 0.08 |
| VES | 147 | 4 | 28 | 9 | 38 | 0.09 |
| CON | 299 | 8 | 59 | 19 | 78 | 0.10 |
| LUE | 57 | 3 | 10 | 3 | 13 | 0.18 |
| *Mean value Silicate soils* | | | | | | **0.12** |

**Table S5:** Contents of microbial C ($C_{mic}$), N ($N_{mic}$), and P ($P_{mic}$), $C_{mic}/P_{mic}$ and $C_{mic}/N_{mic}$ mass ratios as well as mass ratios of organic C ($C_{org}$) over organic P ($P_{org}$) and organic N ($N_{org}$) in the Ah1 horizons of the soils on silicate parent material described in Lang *et al.* (2017). Note that $P_{mic}$ in the soils with silicate parent material in contrast to that in the soils on carbonate parent material has been quantified using the gaseous ("CFE") instead of liquid fumigation ("Resin") method. According to Bergkemper et al. (2016) the amount of $P_{mic}$ yielded from a given soil with the CFE method is two times that yielded with the resin method from the same soil. $P_{mic}$ values of the silicate soils printed in italic fonts in brackets were calculated from the respective $P_{mic}$ value obtained by the CFE method divided by the factor 2 to enable a comparison between silicate and carbonate sites.

| Site | Method | $C_{mic}$ | $N_{mic}$ | $P_{mic}$ | $P_{resin}$ | $C_{mic}/P_{mic}$ | $C_{mic}/N_{mic}$ | $C_{org}/P_{org}$ soil | $C_{org}/N_{org}$ soil | $\frac{P_{mic}/C_{mic}}{P_{org}/C_{org}}$ | $\frac{N_{mic}/C_{mic}}{N_{org}/C_{org}}$ |
|---|---|---|---|---|---|---|---|---|---|---|---|
| | | | | | | | | | | *(Factor of enrichment in soil microorganisms relative to SOM)* | |
| | | --------------------- µg $g^{-1}$ -------------------- | | | | ---------------------- g $g^{-1}$ ----------------------- | | | | | |
| *Bad Brückenau* | CFE | 1223 | 87 | 100 *(50)* | 116 | 12 *(24)* | 14 | 103 | 15.7 | 8.6 *(17)* | 1.1 |
| *Mitterfels* | CFE | 795 | 82 | 111 *(56)* | 70 | 7.2 *(14)* | 10 | 198 | 18.1 | 28 *(56)* | 1.8 |
| *Vessertal* | CFE | 810 | 64 | 79 *(40)* | 40 | 10 *(20)* | 13 | 226 | 17.5 | 23 *(46)* | 1.4 |
| *Conventwald* | CFE | 1392 | 130 | 104 *(52)* | 24 | 13 *(26)* | 11 | 393 | 21.6 | 30 *(60)* | 2.0 |
| *Luess* | CFE | 192 | 12 | 10 *(5)* | 11 | 20 *(40)* | 16 | 905 | 25.7 | 45 *(90)* | 1.6 |

**Table S6:** Activities of phosphomonoesterase [MONO] and phosphodiesterase [DI] as well as phosphomono-esterase/phosphodiesterase ratios in Oe, Oa, and Ah horizons of the profiles on silicate parent material (mean ± standard deviation, n=3).

| | Phosphomonoesterase [MONO] (pH 5.0) | Phosphodiesterase [DI] (pH 8.0 ) | MONO/ DI |
|---|---|---|---|
| | *mg Phenol $g^{-1}$ soil 3 $h^{-1}$* | *mg p-Nitrophenol $g^{-1}$ soil 1 $h^{-1}$* | *ratio* |
| **Oe horizons** | | | |
| *Bad Brückenau* | 41.9 ± 13.3 | 0.15 ± 0.02 | 283 |
| *Mitterfels* | 46.4 ± 2.0 | 0.33 ± 0.05 | 142 |
| *Vessertal* | 93.8 ± 7.5 | 0.33 ± 0.05 | 286 |
| *Conventwald* | 70.6 ± 7.5 | 0.48 ± 0.05 | 149 |
| *Luess* | 55.6 ± 1.5 | 0.41 ± 0.07 | 135 |
| **Oa horizons** | | | |
| *Mitterfels* | 41.4 ± 0.9 | 0.29 ± 0.02 | 142 |
| *Vessertal* | 12.3 ± 0.7 | 0.11± 0.01 | 111 |
| *Conventwald* | 48.5 ± 7.5 | 0.38 ± 0.07 | 127 |
| *Luess* | 20.0 ± 0.4 | 0.08 ± 0.03 | 256 |
| **Ah1 horizons** | | | |
| *Bad Brückenau* | 10.7 ± 0.4 | 0.16 ± 0.00 | 65 |
| *Mitterfels* | 3.8 ± 0.0 | 0.11 ± 0.00 | 34 |
| *Vessertal* | 3.3 ± 0.1 | 0.08 ± 0.00 | 43 |
| *Conventwald* | 7.3 ± 0.3 | 0.24 ± 0.01 | 31 |
| *Luess (EA)* | 1.2 ± 0.0 | 0.01 ± 0.00 | 104 |

**Table S7:** Ecosystem P acquiring and P recycling indicators, Ecosystem P Nutrition Index *ENI*$_P$, and phosphorus ecosystem nutrition strategy of Central European temperate beech forests on sites with different parent material. N1-N7 refer to the ecosystem P acquiring and P recycling indicators presented in Table 3. ND: Not determined. *Normalization to the interval *Bad Brückenau – Lüss* as described in Method Section. Dolostone and limestone soils are ordered according to their stage of pedogenesis. **N6 is not included in *ENI*$_P$ calculation because data are only available for two of eight carbonate sites.

| Site | Acquiring indicators | | | Recycling Indicators | | | | Acquiring indicators | Recycling indicators | Acquiring indicators | Recycling indicators | *ENI*$_P$** | Phosphorus ecosystem nutrition strategy |
|---|---|---|---|---|---|---|---|---|---|---|---|---|---|
| | N1 | N2 | N3 | N4 | N5 | (N6) | N7 | Mean N1-N3 | Mean N4-N7* | Mean normalized* | Mean normalized*,** | | |
| *Dolostone parent material* | | | | | | | | | | | | | |
| Mangfallgebirge N1 | 0.75 | 0.36 | ND | 3.33 | 1.25 | *(0.09)* | 1.27 | 0.55 | 1.95 | 0.18 | 2.13 | -2.0 | **Recycling** |
| Mangfallgebirge N2 | 0.76 | ND | ND | 2.80 | 1.46 | *ND* | 2.49 | 0.76 | 2.25 | 0.56 | 2.51 | -2.0 | **Recycling** |
| Mangfallgebirge S2 | 0.45 | ND | 0.28 | 7.89 | 1.26 | *ND* | 8.52 | 0.36 | 5.89 | -0.17 | 6.90 | -7.1 | **Recycling** |
| Mangfallgebirge S1 | 0.93 | 0.49 | 0.31 | 0.96 | 0.74 | *ND* | 0.94 | 0.57 | 0.88 | 0.22 | 0.86 | -0.6 | Recycling >> Acquiring |
| *Limestone parent material* | | | | | | | | | | | | | |
| Tuttlingen SW | 0.86 | ND | ND | 0.61 | 0.94 | *ND* | 0.33 | 0.86 | 0.63 | 0.75 | 0.55 | 0.2 | Acquiring = Recycling |
| Tuttlingen NE | 0.81 | 0.44 | 0.09 | 0.91 | 0.58 | *(0.14)* | 0.71 | 0.45 | 0.74 | -0.01 | 0.69 | -0.7 | Recycling >> Acquiring |
| Schänis | 0.84 | 0.44 | 0.09 | 0.04 | 1.42 | *ND* | 0.03 | 0.46 | 0.50 | 0.01 | 0.39 | -0.4 | Recycling > Acquiring |
| Bärenthal | 0.87 | ND | ND | 0.07 | ND | *ND* | 0.08 | 0.87 | 0.07 | 0.77 | -0.12 | 0.9 | Acquiring >> Recylcing |
| *Silicate parent material* | | | | | | | | | | | | | |
| Bad Brückenau | **1.00** | **1.00** | **1.00** | 0.05 | 0.31 | *(0.20)* | 0.13 | **1.00** | 0.16 | **1.00** | **0.00** | **1.0** | **Acquiring** |
| Mitterfels | 0.86 | 0.94 | 0.63 | 0.20 | 0.55 | *(0.33)* | 0.36 | 0.81 | 0.37 | 0.65 | 0.25 | 0.4 | Acquiring > Recycling |
| Vessertal | 0.80 | 1.17 | 0.20 | 0.72 | 0.85 | *(0.33)* | ND | 0.72 | 0.78 | 0.49 | 0.74 | -0.3 | Recycling > Acquiring |
| Conventwald | 0.92 | 0.63 | 0.24 | 0.97 | 1.09 | *(0.64)* | 0.91 | 0.60 | 0.99 | 0.26 | 0.99 | -0.7 | Recycling >> Acquiring |
| Lüss | 0.81 | 0.55 | 0.01 | **1.00** | **1.00** | ***(1.00)*** | **1.00** | 0.45 | **1.00** | **0.00** | **1.00** | **-1.0** | **Recycling** |

**Table S8:** Ecosystem P acquiring and P recycling indicators, Ecosystem P Nutrition Index $ENI_P$, and phosphorus ecosystem nutrition strategy of Central European temperate beech forests on sites with different parent material. N1-N7 refer to the ecosystem P acquiring and P recycling indicators presented in Table 3. ND: Not determined. *Normalization to the interval *Bad Brückenau* – *Lüss* as described in Method Section. Dolostone and limestone soils are ordered according to their stage of pedogenesis. **N2, N3, and N6 are not included in $ENI_P$ calculation because data are only available for four (N2, N3) or two (N6) of eight carbonate sites.

| Site | Acquiring indicators | | | Recycling Indicators | | | | Acquiring indicators | Recycling indicators | Acquiring indicators | Recycling indicators | $ENI_P$** | Phosphorus ecosystem nutrition strategy |
|---|---|---|---|---|---|---|---|---|---|---|---|---|---|
| | N1 | (N2) | (N3) | N4 | N5 | (N6) | N7 | Mean N1-N3 | Mean N4-N7* | Mean normalized* | Mean normalized*,** | | |
| *Dolostone parent material* | | | | | | | | | | | | | |
| Mangfallgebirge N1 | 0.75 | *(0.36)* | *ND* | 3.33 | 1.25 | *(0.09)* | 1.27 | 0.75 | 1.95 | -0.33 | 2.13 | -2.5 | **Recycling** |
| Mangfallgebirge N2 | 0.76 | *ND* | *ND* | 2.80 | 1.46 | *ND* | 2.49 | 0.76 | 2.25 | -0.27 | 2.51 | -2.8 | **Recycling** |
| Mangfallgebirge S2 | 0.45 | *ND* | *(0.28)* | 7.89 | 1.26 | *ND* | 8.52 | 0.45 | 5.89 | -1.91 | 6.90 | -8.8 | **Recycling** |
| Mangfallgebirge S1 | 0.93 | *(0.49)* | *(0.31)* | 0.96 | 0.74 | *ND* | 0.94 | 0.93 | 0.88 | 0.63 | 0.86 | -0.2 | Recycling > Acquiring |
| *Limestone parent material* | | | | | | | | | | | | | |
| Tuttlingen SW | 0.86 | *ND* | *ND* | 0.61 | 0.94 | *ND* | 0.33 | 0.86 | 0.63 | 0.29 | 0.55 | -0.3 | Recycling > Acquiring |
| Tuttlingen NE | 0.81 | *(0.44)* | *(0.09)* | 0.91 | 0.58 | *(0.14)* | 0.71 | 0.81 | 0.74 | 0.02 | 0.69 | -0.7 | Recycling >> Acquiring |
| Schänis | 0.84 | *(0.44)* | *(0.09)* | 0.04 | 1.42 | *ND* | 0.03 | 0.84 | 0.50 | 0.18 | 0.39 | -0.2 | Recycling = Acquiring |
| Bärenthal | 0.87 | *ND* | *ND* | 0.07 | ND | *ND* | 0.08 | 0.87 | 0.07 | 0.33 | -0.12 | 0.4 | Acquiring >> Recycling |
| *Silicate parent material* | | | | | | | | | | | | | |
| Bad Brückenau | **1.00** | ***(1.00)*** | ***(1.00)*** | 0.05 | 0.31 | *(0.20)* | 0.13 | **1.00** | 0.16 | **1.00** | **0.00** | **1.0** | **Acquiring** |
| Mitterfels | 0.86 | *(0.94)* | *(0.63)* | 0.20 | 0.55 | *(0.33)* | 0.36 | 0.86 | 0.37 | 0.26 | 0.25 | 0.0 | Acquiring = Recycling |
| Vessertal | 0.80 | *(1.17)* | *(0.20)* | 0.72 | 0.85 | *(0.33)* | ND | 0.80 | 0.78 | -0.07 | 0.74 | -0.8 | Recycling >> Acquiring |
| Conventwald | 0.92 | *(0.63)* | *(0.24)* | 0.97 | 1.09 | *(0.64)* | 0.91 | 0.92 | 0.99 | 0.56 | 0.99 | -0.4 | Recycling > Acquiring |
| Lüss | 0.81 | *(0.55)* | *(0.01)* | **1.00** | **1.00** | ***(1.00)*** | **1.00** | 0.81 | **1.00** | **0.00** | **1.00** | **-1.0** | **Recycling** |

**Figure S1:** Citric acid-extractable (plant-available) soil P stocks at temperate beech forest sites on silicate parent material; data from Lang et al. (2017). Shown are absolute and relative contributions of organic and inorganic P. For a detailed description of sites, please read caption of Fig. 2 in main paper.

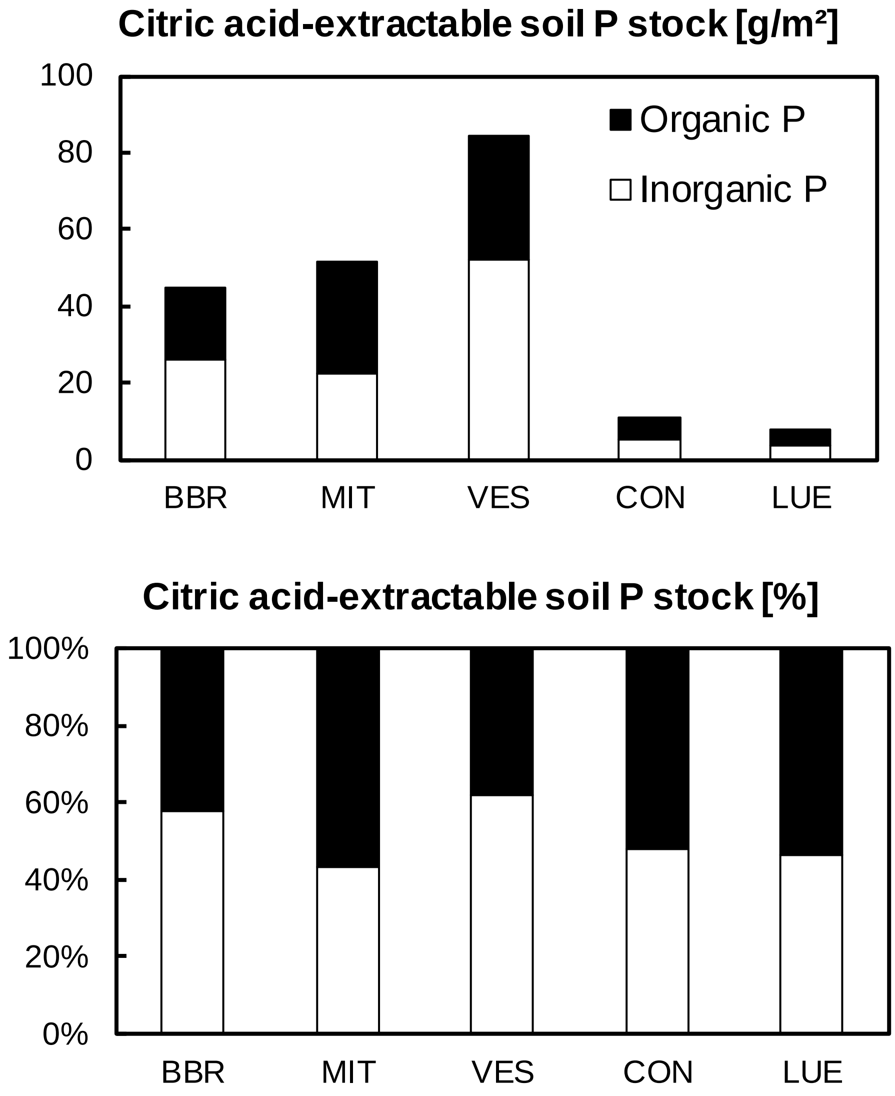

**Figure S2:** Microbial biomass carbon (MBC) and extractable organic carbon (EOC) for a) *Mangfallgebirge N1*, b) *Tuttlingen NW*, and c) *Schänis*. Significant differences between horizons are denoted with lower-case letters at $p$<0.05.

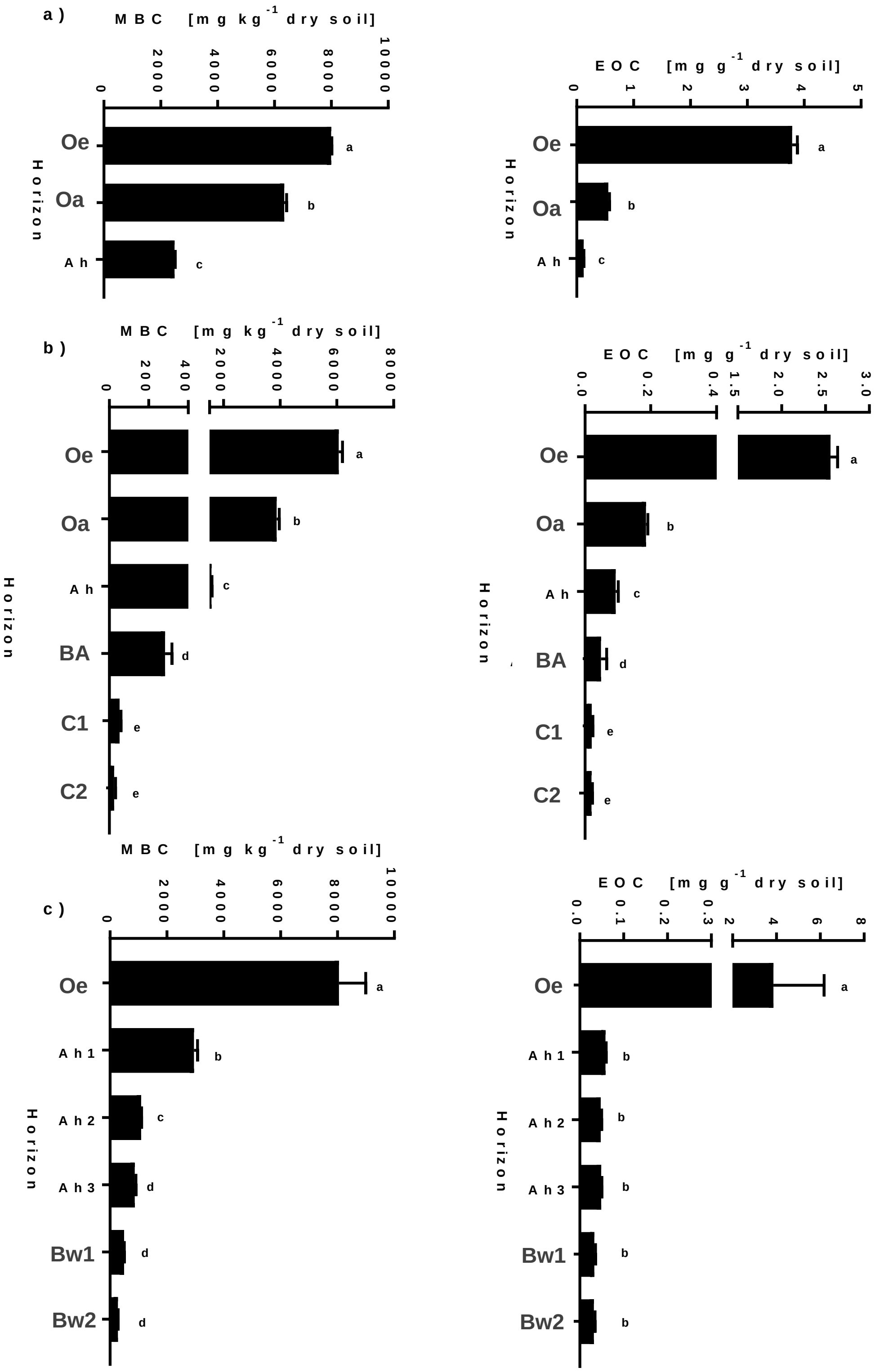

**Figure S3:** Microbial biomass nitrogen (MBN) and extractable nitrogen (EN) for a) *Mangfallgebirge N1*, b) *Tuttlingen NW*, and c) *Schänis*. Significant differences between horizons are denoted with lower-case letters at $p<0.05$.

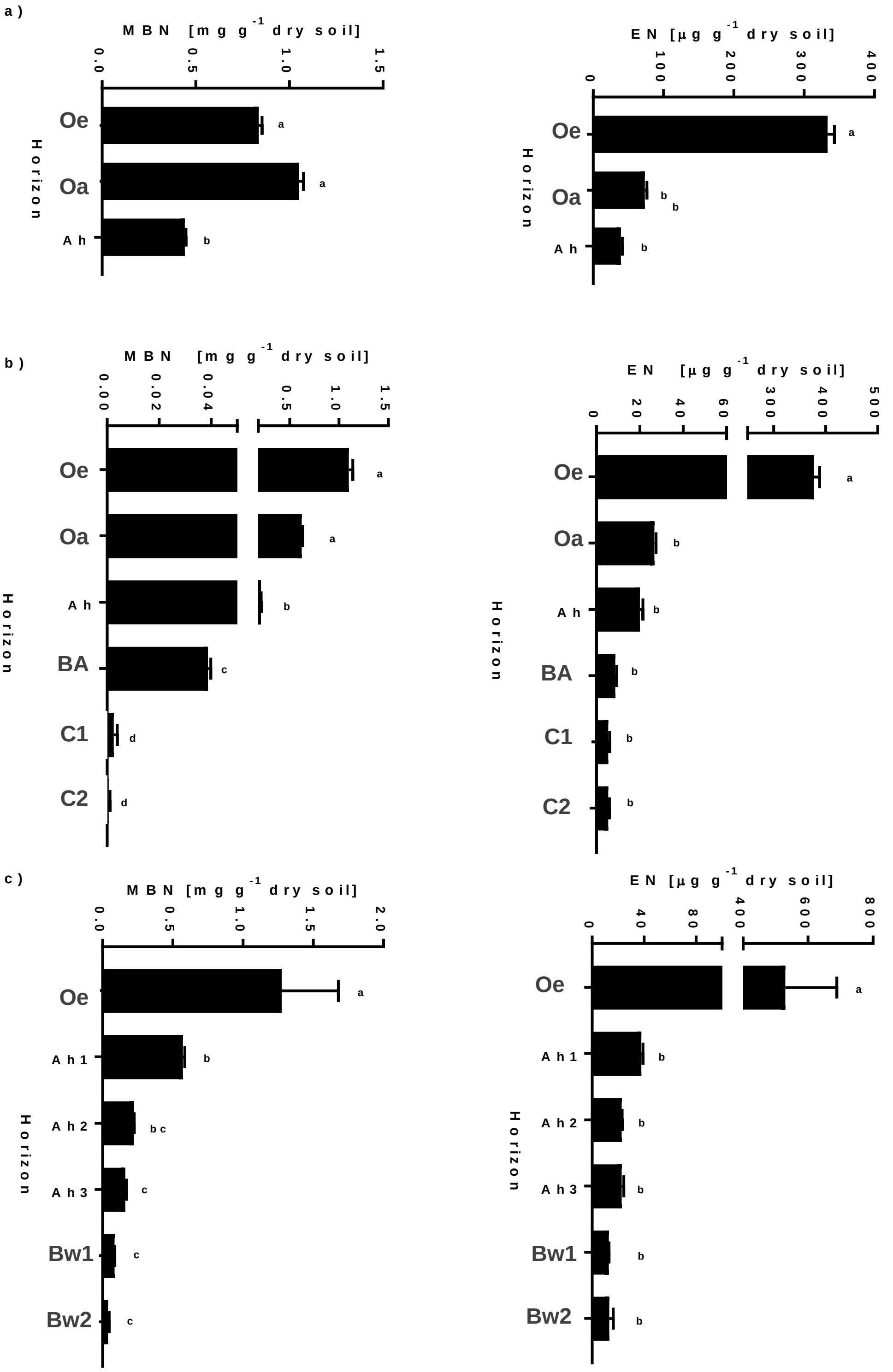

**Figure S4:** Contents of total Fe, total P, and P/Fe mass ratio in the profiles *Tuttlingen NE, Mangfallgebirge S1, Bärenthal, and Schänis.*

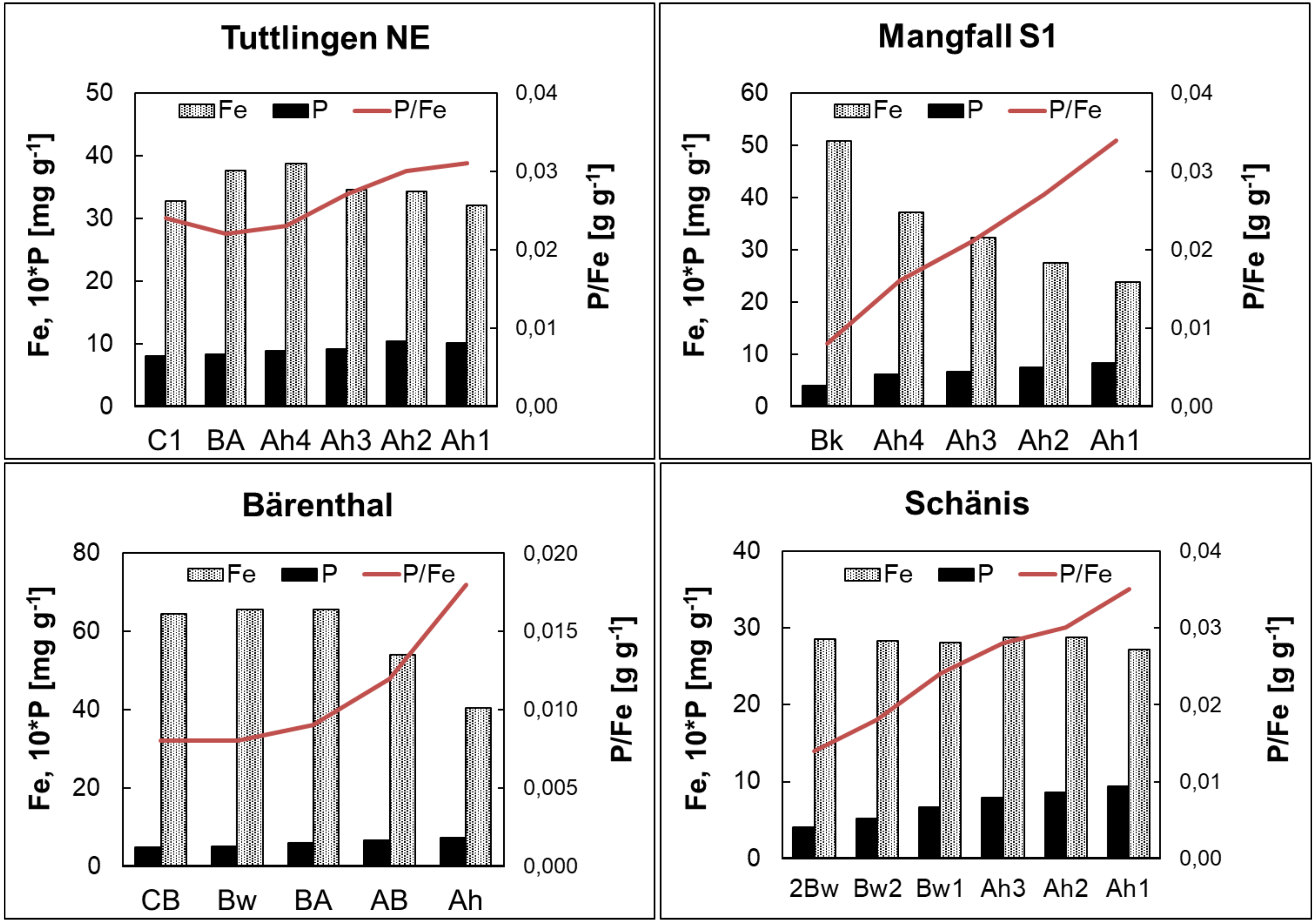

Supplement: Supplementary file 1 — Supplementary file1 (PDF 840 KB) [file 10533_2021_884_MOESM1_ESM.pdf]
